# Advancing censored geochemical Au prediction through Bayesian spatial models and Random Forest with fractal-based background separation

**DOI:** 10.1038/s41598-026-34999-4

**Published:** 2026-01-06

**Authors:** Hossein Mahdiyanfar

**Affiliations:** Department of Mining Engineering, University of Gonabad, Gonabad, Iran

**Keywords:** Censored geochemical data, Bayesian Gaussian Random Field (BGRF), Random Forest (RF), Fractal analysis, Spatial modeling, Environmental sciences, Solid Earth sciences

## Abstract

Censored geochemical data, particularly below detection limits, challenge mineral exploration by biasing anomaly delineation and spatial patterns. This study presents a multi-stage framework combining Bayesian Gaussian Random Field (BGRF) modeling with Random Forest (RF) learning, enhanced by fractal-based background separation, to accurately predict censored Au concentrations. 14 samples with gold concentrations below 5 ppb were hypothesized as censored data to enable a more accurate evaluation of the model’s performance based on their real Au concentrations. Unlike constant substitution methods, the framework preserves censored information and reconstructs spatial variability through probabilistic inference and nonlinear learning. The BGRF model incorporates spatial coordinates and Cu as the principal covariate to capture spatial autocorrelation and inter-element associations, producing probabilistic estimates for hypothesized censored data (HCD) that are then used to train the RF under a 5-fold out-of-fold scheme. The HCD estimated by spatial BGRF covariate model were performed as inputs for RF prediction model. A targeted calibration and scaling procedure reduces detection-limit bias and improves low-range predictions. Comparative analyses show that the calibrated and scaled RF–BGRF model substantially enhances accuracy and preserves realistic geochemical structures, outperforming half the detection limit (LD-half) or the detection limit divided by the square root of two (LD-rad2) approaches. This framework offers a promising tool for refining left-censored geochemical data in complex geological environments.

## Introduction

Geochemical data serve as a fundamental tool in mineral exploration and resource modeling, providing essential information for understanding elemental distributions and identifying geochemical anomalies. In recent years, machine learning approaches have garnered increasing attention in geochemical studies, offering enhanced capabilities for predicting element concentrations and modeling complex spatial patterns compared to traditional techniques^1^. A persistent challenge in geochemical datasets is the presence of censored values, where measured elemental concentrations fall below the detection limits of analytical instruments. This issue, combined with spatial heterogeneity, multimodality, and background noise, complicates statistical modeling and may obscure subtle natural variability^2^. Proper handling of such data is critical to preserve true variance, accurately capture spatial and statistical variations, and prevent distortion of the data distribution^3^. Conventional methods for managing censored geochemical data typically involve either removing non-detect samples or substituting them with fixed values, such as half the detection limit (LD-half) or the detection limit divided by the square root of two (LD-rad2). While computationally simple and widely used, these approaches inherently reduce variance, excessively smooth the data, and fail to capture subtle local variations among censored observations. Consequently, although overall mean values may remain approximately preserved, underlying statistical and spatial patterns are often inadequately represented^3^. To overcome these limitations, advanced statistical frameworks and machine learning techniques have been increasingly applied to censored geochemical datasets. Constant-value substitution methods distort the true shape of the distribution particularly in the lower range and should be replaced by approaches that reconstruct the actual behavior of the data within that range rather than imputing an arbitrary constant^4^. Carranza^5^ described that in geochemical exploration, the presence of censored values often distorts the statistical distribution of data, which can lead to biased anomaly delineation. He demonstrated that applying logratio transformation to stream sediment data with censored values improves the detection and mapping of geochemical anomalies by reducing compositional bias.

Recent advances in statistical modeling have highlighted the importance of probabilistic approaches in dealing with censored geochemical data. Suzuki et al.^6^ applied a Bayesian estimation framework using Markov Chain Monte Carlo (MCMC) simulations to address left-censored datasets, demonstrating its effectiveness in reducing uncertainty and providing more reliable parameter estimates. In particular, Bayesian Gaussian random field models employing MCMC sampling have been developed to analyze censored spatial data, offering accurate estimation under complex spatial constraints^7^. Bayesian models, spatial models, and tree-based algorithms such as Random Forest and Quantile Regression Forest enable prediction of censored values while accounting for spatial dependencies, uncertainties, and inherent data variability^8,9^. Computationally scalable approaches, including Bayesian SPDE models and Gaussian Markov random fields, provide efficient solutions for large spatially censored geochemical datasets^10^. Tadayon^11^ employed a Bayesian framework that accounts for non-Gaussian distributions to estimate parameters for censored spatial data. Ellefsen et al.^12^ applied a probabilistic hierarchical modeling approach to sparse geochemical datasets, allowing the incorporation of measurements with different detection limits.

Machine learning techniques have also been adapted to address challenges in censored geochemical data. For instance, nearest-neighbor Gaussian process models integrate spatial information with sparse approximations to accurately predict censored values in multi-scale, heterogeneous geochemical environments^13^. Despite these advances, many previous studies have focused on single frameworks or relied on simplified statistical assumptions, often excluding censored observations. In this study, we propose an innovative multi-stage framework for the effective management of censored geochemical data. Our approach integrates spatial Bayesian Gaussian modeling with Random Forest algorithms, enabling accurate reconstruction of spatial correlation structures while leveraging nonlinear learning to capture complex patterns in censored data. In addition, fractal-based background separation effectively distinguishes mineral signals from background variability, a feature rarely addressed in prior research. Fractal-based background separation has emerged as a powerful and increasingly important approach in geochemical exploration, offering an objective and data-driven means of distinguishing background variability from mineralization-related anomalies, particularly in datasets affected by left-censoring. The concentration–number (C–N) fractal model delineates threshold values through scale-dependent changes in the slope of the log–log N–C relationship, thereby enabling robust identification of geochemical populations in heterogeneous or multimodal environments^14^. In contrast to conventional statistical thresholds, which are often sensitive to outliers or distributional assumptions, fractal models provide more reliable handling of non-Gaussian geochemical data while preserving subtle background structures. Recent studies have further demonstrated the effectiveness of fractal thresholds for anomaly mapping in complex terrains and for enhancing the interpretability of low-grade geochemical signals^15–17^. Incorporating fractal-based background separation thus establishes a rigorous foundation for subsequent spatial modeling and machine-learning workflows, ensuring that predictive models are trained on geochemically coherent and statistically consistent background populations. Random Forest algorithms, in particular, provide robust predictive capabilities, capturing complex variable relationships and enhancing overall modeling accuracy^18^.

A key advantage of the proposed framework is its ability to preserve censored information for predictive purposes without requiring deletion or substitution. Moreover, the use of robust statistical metrics, including Bayesian predictive density distributions and mean-based evaluations, ensures that predictions are statistically defensible and highly reliable. By combining spatial modeling, machine learning, and fractal-based background separation, this multi-layered framework provides a comprehensive and innovative approach for analyzing left-censored geochemical data, offering improved predictive accuracy, enhanced generalizability, and superior representation of natural geochemical variability. Importantly, while simple substitution methods such as LD-half and LD-rad2 remain convenient, they frequently suppress variance and introduce systematic biases, limiting their effectiveness in modern geochemical exploration.

## Material and methods

### Case study and data set

The study area is located in Northern Dalli, which hosts porphyry Cu–Au mineralization and lies approximately 70 km from the city of Arak. The investigated zone covers more than 350 × 400 m². It is situated within the Urmieh–Dokhtar Magmatic Arc (UDMA), a well-known metallogenic belt in Central Iran that contains several major porphyry copper deposits and is genetically associated with Neo-Tethyan magmatism^19^. This magmatic arc is part of the Central Iranian block and is composed mainly of calc-alkaline intrusive bodies (stocks) together with volcanic rocks belonging to the Sahand–Bazman belt^20^. Accordingly, Northern Dalli represents a segment of one of the most significant volcanic–plutonic and metallogenic provinces of Central Iran.

**Fig. 1:**
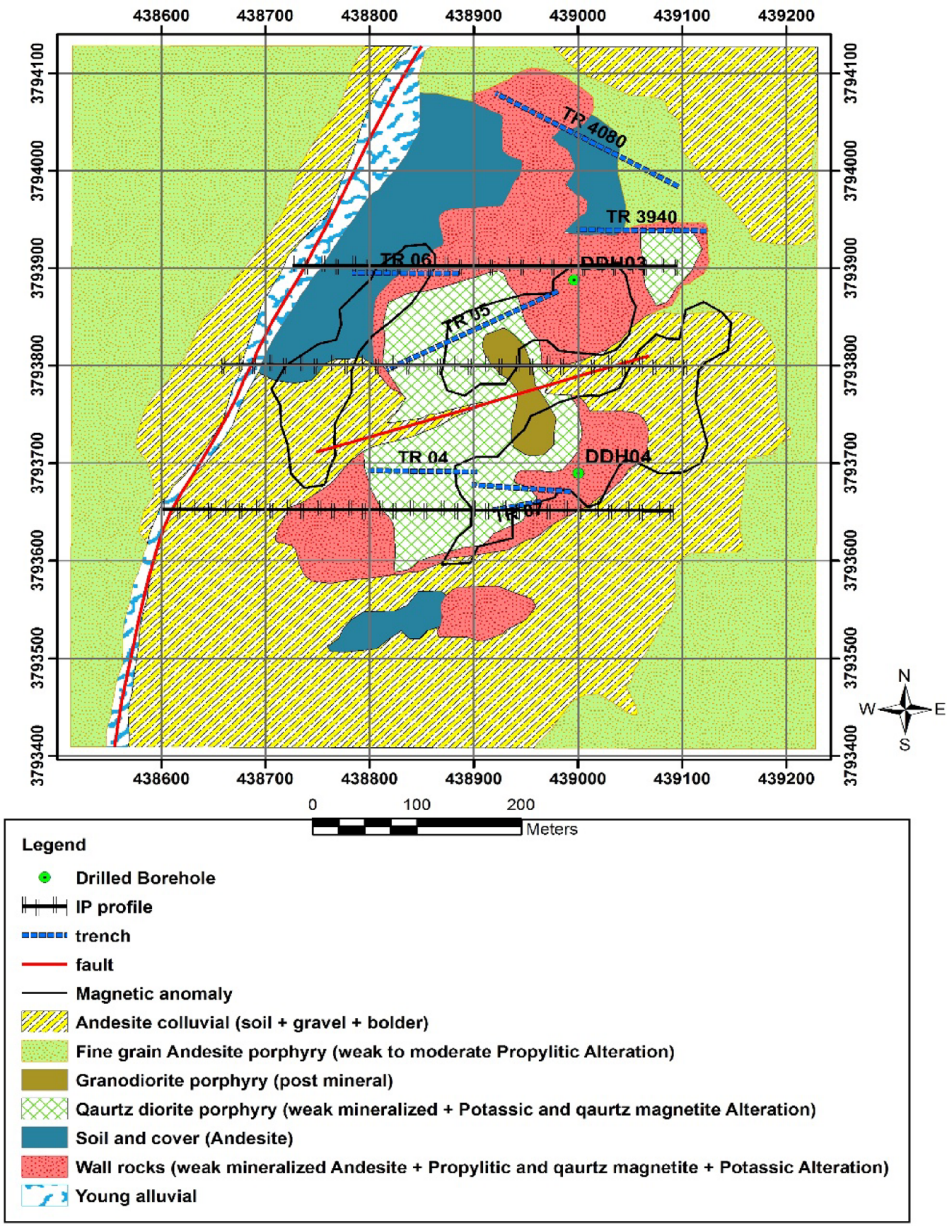
Geological map of the northern part of Dalli area.

Local geology indicates that the mineralized host rocks at Northern Dalli consist of calc-alkaline plutonic units and subvolcanic intrusions. Volcaniclastic sequences, amphibole–dacitic andesite porphyry, and pyroclastic rocks are associated with Late Miocene stratovolcanic successions, which extend for nearly 30 km along a NNE trend. The mineralization mainly occurs within quartz diorite porphyry intrusions emplaced into andesitic wall rocks^21^. A simplified geological map of the area is presented in Fig. 1.

For this study, 165 soil samples were systematically collected on a 50 × 50 m grid. The < 200 mesh fraction was selected for chemical analysis to ensure that fine particles accurately represent the geochemical conditions. Analyses were carried out at AMDEL Laboratory (Australia) using inductively coupled plasma–mass spectrometry (ICP–MS). The detection limit for gold was reported as 2 ppb, allowing reliable recognition of subtle geochemical anomalies and precise delineation of mineralized zones. The integration of high-resolution sampling, accurate ICP–MS analyses, and well-distributed spatial data provides a robust foundation for identifying and modeling geochemical patterns through advanced machine learning algorithms and fractal-based techniques.

**Fig. 2:**
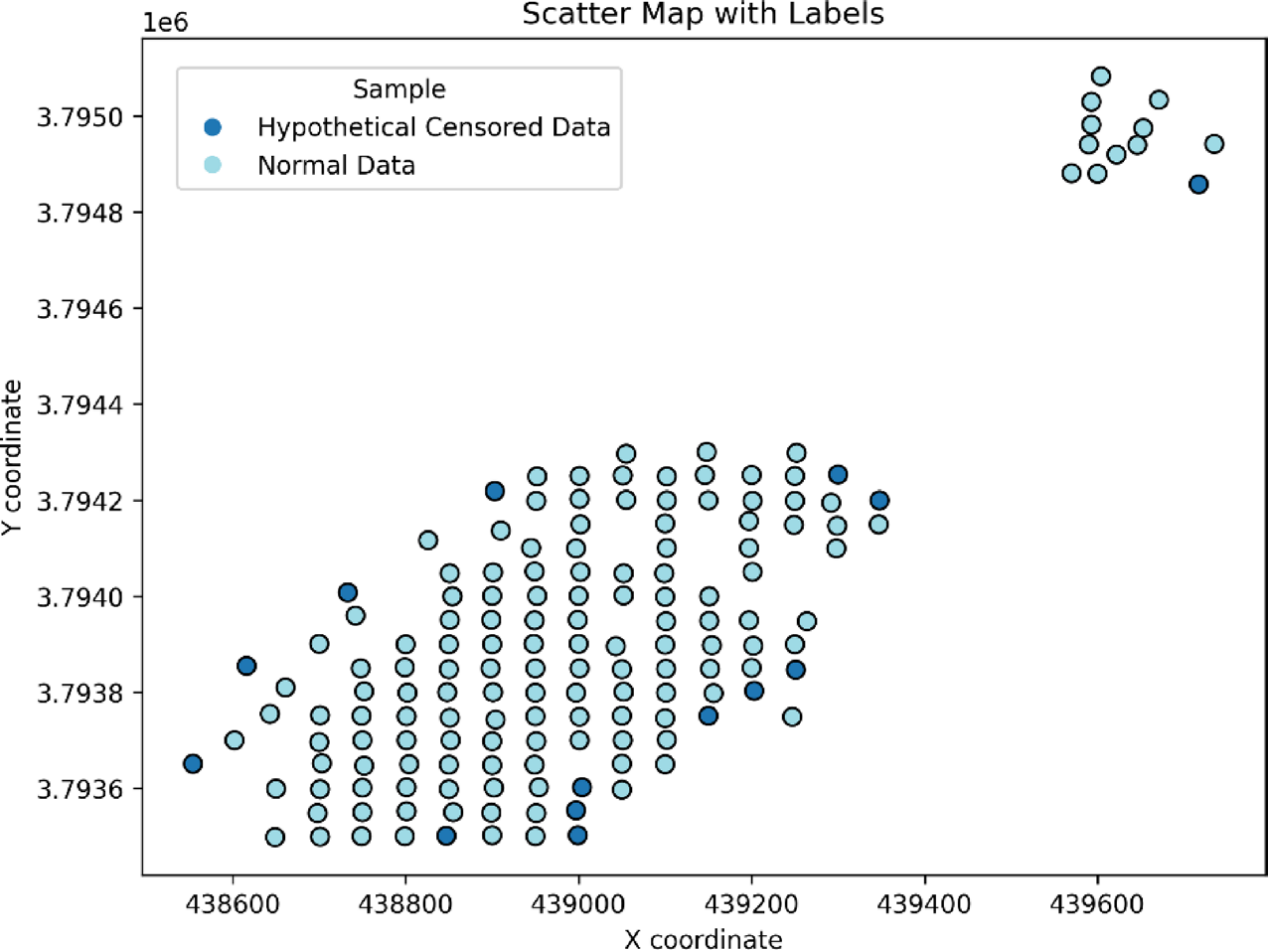
The spatial distribution map of geochemical samples including HCD and normal samples in the studied area.

In this study, the detection limit for gold was assumed to be 5 ppb, and samples with concentrations below this threshold were treated as hypothetical censored data (HCD). Accordingly, 14 samples were classified as censored. These censored values were predicted using a hybrid RF–BGRF approach, based on the fractal model of gold. Figure 2 shows the distribution of the sampling points along with the locations of censored and uncensored samples. The censored samples are mainly located at the margins of the sampling grid and within the background domain. This distribution is consistent with the actual mineralization pattern of the area, indicating that the censored values are unlikely to result from analytical or sampling errors and most probably represent genuinely low grades. Therefore, these data were used to evaluate the reliability and predictive performance of the proposed hybrid method.

**Fig. 3:**
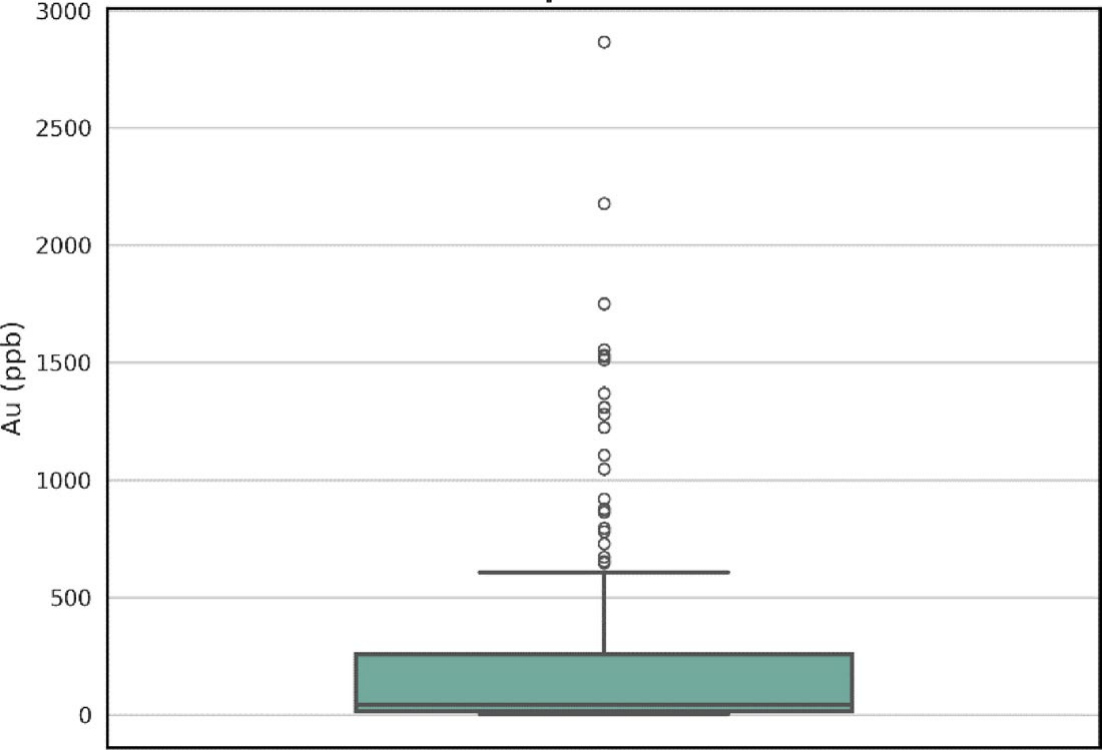
The boxplot of Au element for all of data.

A box plot of gold concentrations is presented in Fig. 3. As observed, a considerable number of samples were identified as outliers, which are associated with the anomalous population. Since the fractal model was employed to distinguish background samples, the prediction was performed solely on the geochemical background population, and outliers belonging to the anomalous domain were excluded from the training dataset. The dataset initially contained analytical results for 30 elements. Among these, five elements showing the highest absolute correlation with gold in the background population, as determined by the fractal model, were selected as predictor variables. Au concentrations of HCD were then predicted based on these selected features.

Table 1 reports the fundamental descriptive statistics for the six geochemical elements (Au, Cu, Fe, Ni, Ti, and B) that were used within the machine-learning component of the proposed framework. These statistics provide an overview of the range, central tendency, dispersion, and distributional properties of each variable, supporting a clearer understanding of their behavior prior to modeling and aiding in the interpretation of the predictive results.

**Table 1:** Summary statistics of the six key geochemical elements used in the machine learning framework.

	Min	Max	Mean	Median	std	Variance	Skewness	Kurtosis
Cu	49.00	3590.00	584.60	258.00	659.14	434466.92	1.77	3.46
Fe	2.40	6.75	4.54	4.52	0.72	0.52	0.07	0.33
Ni	8.00	68.00	32.23	29.00	14.12	199.42	0.53	-0.73
Ti	298.00	3011.00	1081.09	989.00	511.25	261380.51	1.52	3.01
Au	2.00	920.00	143.62	36.00	216.32	46795.56	2.01	3.39
B	4.00	24.00	13.97	14.00	5.02	25.23	0.16	-0.95

### Concentration-number (C-N) fractal analysis

To separate geochemical anomalies of gold from the background, theC-N Fractal method was employed. This approach is based on analyzing the statistical behavior of the element’s concentration distribution and the corresponding number of samples for each concentration level. It is particularly reliable in complex, multimodal environments and in datasets containing left-censored values. In this method, the concentration data are first arranged in ascending order. For each concentration value, the number of samples with concentrations equal to or greater than that value is then calculated. The fractal relationship between the number of samples and the element concentration is defined as follows:

1 $$\:\mathrm{N}\left(\mathrm{C}\right)=\mathrm{k}\cdot\:{C}^{-D}$$

Here, N(C) represents the number of samples with concentrations equal to or greater than C, k is the scaling constant, and D denotes the fractal dimension. The fractal log–log plot typically consists of several linear segments with different slopes. Breaks between these segments indicate changes in the geological processes controlling element concentrations and are considered as thresholds for identifying geochemical anomalies^22,23^; Ghannadpour et al., 2024; Safari et al.^24^. These breaks typically appear as slope-change points in the log–log plot and can be identified using piecewise linear fitting or logarithmic derivative analysis. The segment with a gentler slope represents the geochemical background, whereas the steeper segment corresponds to mineralized anomalies. In datasets containing left-censored values, this method utilizes the underlying distribution of the censored data to extract anomaly thresholds instead of discarding values below the detection limit^25^.

In the present study, thresholds derived from the C-N Fractal method were employed as the basis for separating background and anomalous data during the preprocessing stage. The geochemical background population was then used as input for Bayesian spatial models and machine learning algorithms to enhance predictive accuracy.

### Bayesian Gaussian Random Field (BGRF)

The Bayesian Gaussian Random Field (BGRF) model provides a hierarchical Bayesian structure for analyzing spatial data, where the response variable is treated as a stochastic process and spatial dependence between sampling locations is captured through a covariance function^26^. In this study, in addition to spatial coordinates, copper concentration was incorporated as a covariate to account for geological co-association and enhance predictive accuracy. This choice improves anomaly delineation and strengthens the model’s ability to distinguish mineralized zones from background variation. The observed concentration at location s_i_ is modeled as:

2 $$y\left( {s_{i} } \right) = x\left( {s_{i} } \right)^{{ \top }} \beta + w\left( {s_{i} } \right) + \varepsilon \left( {s_{i} } \right)$$

Where y(s_i_) is goal variable (Au concentration) at location s_i_, x(s_i_) is vector of covariates including spatial coordinates and element (Cu) concentration, β regression coefficients, w(s_i_) is spatial random effect modeled as a Gaussian process, and ε(s_i_) is independent Gaussian noise. The spatial effect w(s) is defined as a zero-mean Gaussian process with covariance function C(s, s′;ϕ):

3 $$w(s)\sim GP(0,C(s,{\text{ }}s';\phi ))$$

Where ϕ is the set of covariance parameters. A commonly used exponential covariance function is:

4 $$\:\mathrm{C}(\mathrm{s},{\mathrm{s}}^{{\prime\:}},{\upvarphi\:})={{\upsigma\:}}^{2}\mathrm{e}\mathrm{x}\mathrm{p}(-\parallel\:\mathrm{s}-\mathrm{s}{\prime\:}\parallel\:/{\uprho\:})$$

Where σ^2^ is the spatial variance and ρ is the range parameter controlling spatial correlation^26^. The posterior distribution is then obtained and for left-censored data (i.e., values below detection limit), the likelihood is modified as:

5 $$P(y\left( {s_{i} } \right) < L) = \Phi \left( {\left( {L - x\left( {s_{i} } \right)^{{ \top }} \beta - w\left( {s_{i} } \right)} \right)/\tau } \right)$$

Where Φ(⋅) is the cumulative distribution function of the standard normal distribution^7^. This formulation allows censored observations to contribute to inference without imputation or deletion. Posterior inference was performed using Markov Chain Monte Carlo (MCMC) sampling. For large datasets, scalable approximations such as Stochastic Partial Differential Equations (SPDE) and Gaussian Markov Random Fields (GMRF) were used to reduce computational complexity^10,27^.

### Random Forest algorithm with K-fold cross-validation and out-of-fold estimation

In this study, the Random Forest (RF) method was employed to predict geochemical HCD of Au concentrations. RF is a nonparametric ensemble method that builds many decision trees and aggregates their outputs to improve predictive stability and mitigate overfitting^28^. Its capability to model nonlinear relationships, manage high-dimensional inputs, and remain robust under noisy conditions renders it highly suitable for mineral exploration tasks characterized by complex geochemical patterns^29^. A stratified k-fold cross-validation scheme was adopted to promote robust generalization and avoid optimistic bias. The dataset was partitioned into k folds with preserved class proportions; in each iteration, one fold served as the validation set while the remaining folds formed the training set, ensuring each observation was validated exactly once. Out-of-fold (OOF) predictions were harvested by using, for each sample, only the model trained on folds that did not include that sample. This approach yields an unbiased estimate of performance, eliminating data leakage between training and validation sets, and is especially valuable for ensemble stacking or meta-learning frameworks^30^. Key hyper parameters including the number of trees, maximum tree depth, and minimum samples per leaf were tuned via grid search within the cross-validation framework.

## Results and discussion

**Fig. 4:**
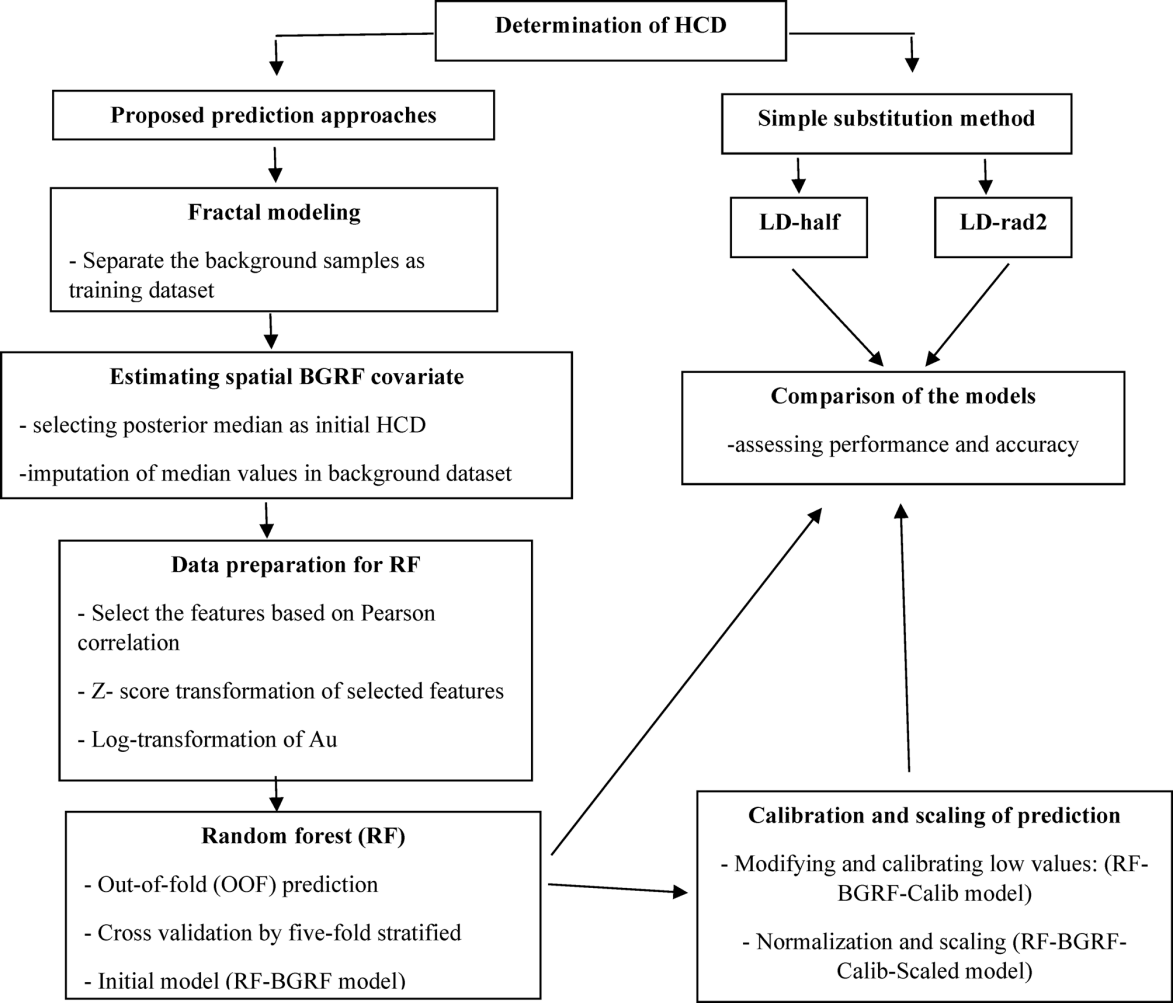
Flowchart of the proposed multi-stage methodology for predicting HCD for Au element in geochemical data.

Before proceeding to numerical analyses and model performance evaluation, it is essential to outline the proposed methodological framework step by step. In this study, a multi-stage approach was developed to predict censored gold (Au) values in geochemical data. The workflow includes background separation using the fractal method, spatial modeling through the Bayesian Gaussian Random Field (BGRF), and machine learning implementation via the Random Forest (RF) algorithm. This framework was designed to accurately capture spatial structures, account for inter-element relationships, and reduce errors associated with values below the detection limit. The flowchart illustrated in Fig. 4 shows the proposed hybrid approach and main stages of the applied process in this investigation.

### Fractal analysis and geochemical background identification

In this study, the C-N Fractal method was employed to accurately separate background samples from geochemical anomalies for Au element. This approach was selected due to its high capability in detecting subtle variations in background data and preventing over prediction in subsequent predictive modeling. The fractal analysis is particularly important for handling censored data, as it utilizes the underlying distribution of values below the detection limit to define anomaly thresholds instead of discarding them. This ensures that essential background information is preserved and important geochemical signals are not lost.

**Fig. 5:**
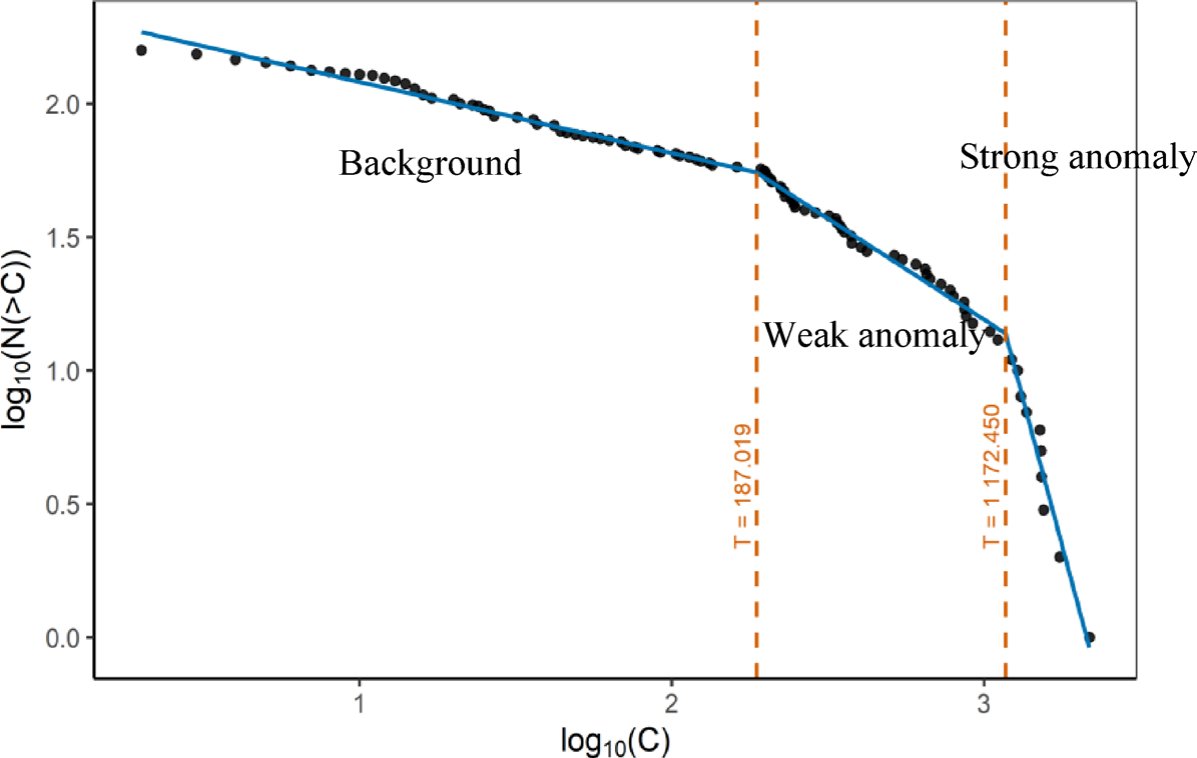
N-C Fractal plot for Au showing the two primary thresholds (187 and 1172) and classification into background, weak anomaly, and strong anomaly.

**Fig. 6:**
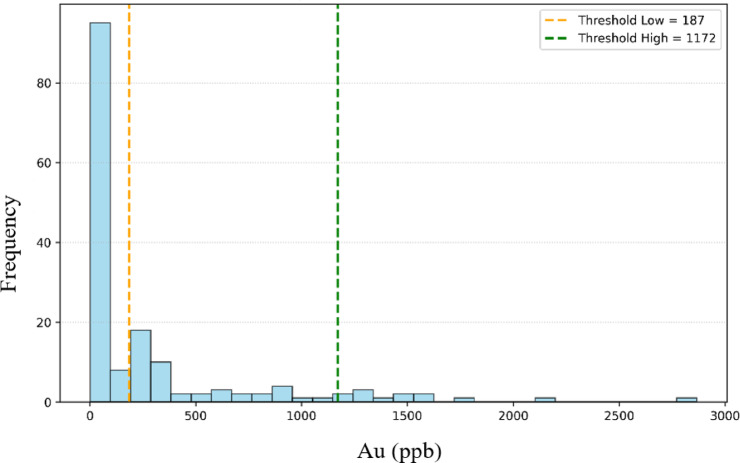
Histogram of all samples for Au element with indicated fractal thresholds for background and anomalous populations.

**Fig. 7:**
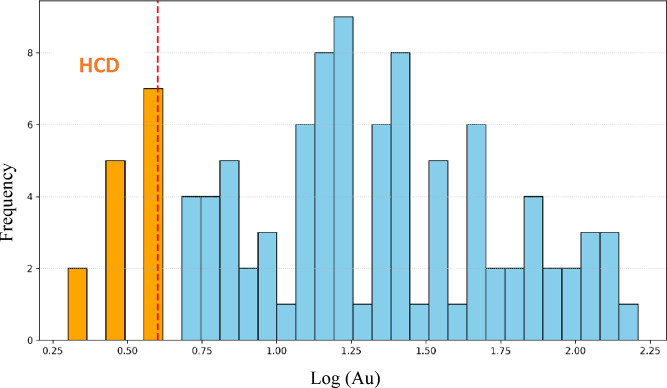
Log-transformed histogram of Au concentrations in the background population derived from the fractal modeling, showing the positions of hypothetical censored data (HCD).

The breakpoints in the C–N fractal plot were determined objectively using a statistical segmented regression approach rather than by visual inspection. Specifically, we applied the piecewise linear regression algorithm implemented in the segmented package in R to the log–log transformed concentration–number data. Consistent with established geochemical theory, we specified two breakpoints a priori (resulting in three classes: background, weak anomaly, and strong anomaly). The location of these two breakpoints was estimated statistically by the model through iterative maximum likelihood estimation, starting from initial guesses at the one-third and two-third quantiles of the logarithmic data. The algorithm iteratively refined breakpoint positions to minimize the residual sum of squares for the segmented model and maximize overall fit, stopping when convergence criteria were met. For each breakpoint, the model also returned an estimated standard error. The results of the fractal analysis for gold are presented in Fig. 5. Two primary thresholds, 187 and 1172, were identified on the fractal plot, classifying the dataset into three categories: background, weak anomaly, and strong anomaly populations. Only background values were selected for model training to minimize overfitting and to retain the inherent geochemical patterns. Figure 6 shows the histogram of all Au data with the threshold locations, and Fig. 7 presents the histogram of the log-transformed background Au values with the HCD indicated. These visualizations confirm the accurate separation of background and anomalous populations and provide a robust basis for precise prediction.

**Fig. 8:**
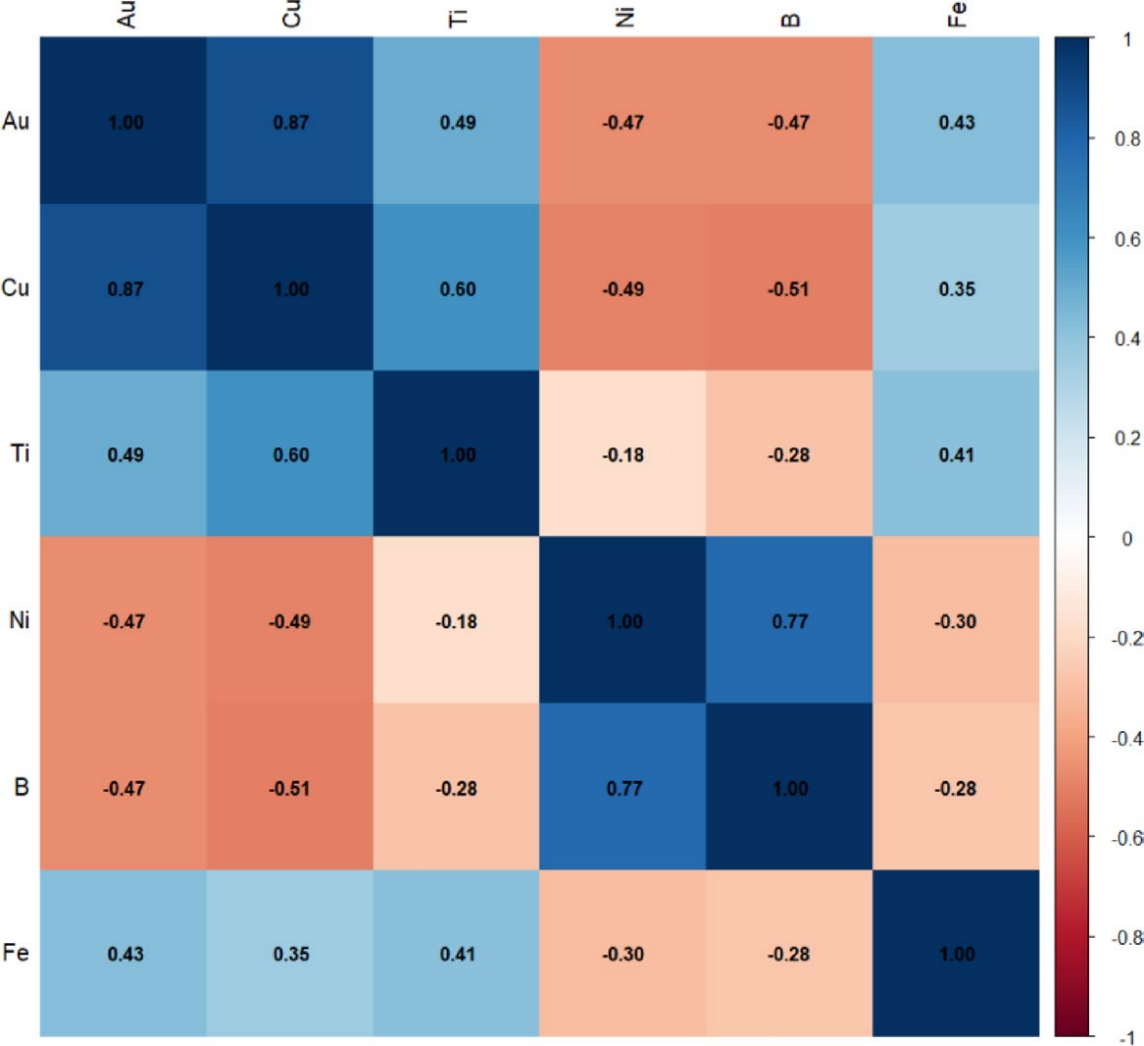
The heat map of Pearson correlation of selected elements with Au based on geochemical background samples.

For predicting HCD gold values, five elements exhibiting the highest absolute Pearson correlation with gold in the background population were selected as predictor variables. The Pearson correlation matrix for these elements is shown in Fig. 8, indicating that Cu has the strongest correlation with Au (*r* = 0.87). The other selected elements include Ti, B, Ni, and Fe. Choosing these features based on their correlation in the background population ensures that real geochemical patterns and inter-element relationships are maintained during the prediction process, allowing the model to estimate censored values more accurately. The application of the fractal method in this study not only enabled precise identification of the background population but also played a crucial role in enhancing the prediction of censored data. Preserving authentic background samples, reducing noise from anomalous data, and selecting relevant predictor features allowed Bayesian spatial models and machine learning algorithms to better learn complex inter-element relationships and produce more reliable predictions. Moreover, this approach mitigates over prediction and improves the prediction of HCD with higher confidence.

### Bayesian Gaussian Random Field (BGRF)

In this study, the prediction of gold HCD values were performed using a model of spatial BGRF with covariate. The primary objective of BGRF is to generate probabilistic estimates for censored samples with values below the detection limit (0–5 ppb), while simultaneously accounting for spatial and inter-element dependencies. Unlike traditional substitution methods with fixed values, BGRF explicitly models uncertainty and provides a 95% confidence interval for each low-grade sample.

The input dataset was divided into censored and non-censored samples, and a latent variable was defined for the censored observations within the 0–5 ppb range to estimate their true values probabilistically. The model incorporated two key predictors: standardized spatial coordinates (X, Y) and the standardized Cu concentration, which exhibited the highest correlation with Au in the background population. The simultaneous use of spatial coordinates and Cu allows the model to capture both local spatial variations and inter-element relationships, thereby preserving the subtle geochemical patterns of the background population more accurately. Prior to modeling, both the spatial coordinates and Cu values were standardized to ensure comparability and improve model performance. Standardization eliminates scale differences between features, preventing any single variable from dominating the model, and enhances MCMC convergence, leading to more stable posterior sampling and predictive estimates.

The BGRF model consists of three main components: a linear regression based on selected features (coordinates and Cu), a latent Gaussian random field to account for spatial autocorrelation and preserve local variations, and a model error term modeled with a Half Normal distribution to explicitly represent uncertainty. The selection of Cu as a key covariate in Au predictive modeling, beyond its observed strong statistical correlation, has robust roots in the geological context of the study area the porphyry Cu-Au mineralization at Northern Dalli. In porphyry environments, Au and Cu are frequently transported and deposited by common ore-forming hydrothermal fluids, consequently exhibiting a close spatial and geochemical association. This genetic relationship implies that the spatial distribution patterns of both elements, even at low or censored Au concentrations, are intrinsically interdependent. Therefore, incorporating Cu as a covariate allows for a better elucidation of prevailing mineralization processes and a more accurate reconstruction of true Au patterns, particularly in censored data. This elevates the model from merely a statistical tool to a powerful geoscientific interpretive instrument.

Posterior sampling was conducted with 2000 iterations and 1000 tuning steps, and the posterior predictive distribution for the latent variables was extracted. Posterior medians and 95% confidence intervals were calculated for each censored sample to facilitate comparison with observed values. The BGRF model applied in this study is a spatial regression framework that incorporates both spatially structured random effects and independent Gaussian noise alongside covariates. In this study, the regression coefficients β were defined based on standardized predictors, and following Bayesian practice, it was assumed that these coefficients follow a normal distribution with mean zero and variance ten (β∼Normal(0,10)). This distributional prior, which is widely adopted in statistical modeling, ensures that the estimates remain mathematically stable and statistically unbiased under standardized scaling. Accordingly, the application of this prior not only contributes to the regularization of parameter estimates but also provides a defensible and consistent framework for inference within Bayesian models.

In addition, the independent noise standard deviation σ was assigned a half-normal prior with scale parameter one (σ∼HalfNormal(1)), reflecting the assumption of non-negative variability in residual noise. The spatial length-scale parameter ℓ was modeled with a gamma prior (ℓ∼Gamma(2,1)), which controls the decay rate of spatial correlation and thereby governs the smoothness of the random field. Finally, the amplitude parameter η was given a half-normal prior (η∼HalfNormal(1)), representing the marginal variance of the spatial random field and ensuring non-negative scaling of spatial variability. Together, these priors provide a coherent Bayesian framework that balances flexibility with physical interpretability in the modeling of censored geochemical data. An exponentiated covariance function was employed. This kernel was selected due to its smoothness and its ability to effectively represent long-range spatial continuity in geochemical datasets, especially when gradual variations are expected. All covariates were standardized (Z-score transformation) prior to modeling to ensure comparable scales, prevent dominance by any single predictor, and improve MCMC convergence stability. Samples with Au concentrations ≤ 5 ppb were modeled using a latent variable with a Truncated Normal likelihood. This approach allows censored observations to contribute to posterior inference without constant substitution methods or deletion. The stochastic partial differential equation (SPDE) approximation was not applied in this analysis, since the dataset size was moderate and full MCMC inference for the BGRF model was computationally feasible. The diagnostic indices of the MCMC chains, including the potential scale reduction factor and the effective sample size (ESS), were examined for the parameters β, σ, ℓ, and η. All parameters exhibited acceptable convergence, and the chains remained within the normal range. Convergence diagnostics confirmed stable sampling performance, with all potential scale reduction factors between 1 and 1.04 and bulk effective sample sizes (ESS) ranging from approximately 850 to 1350 across parameters. Visual inspection of trace plots confirmed adequate chain mixing without divergent transitions.

**Fig. 9:**
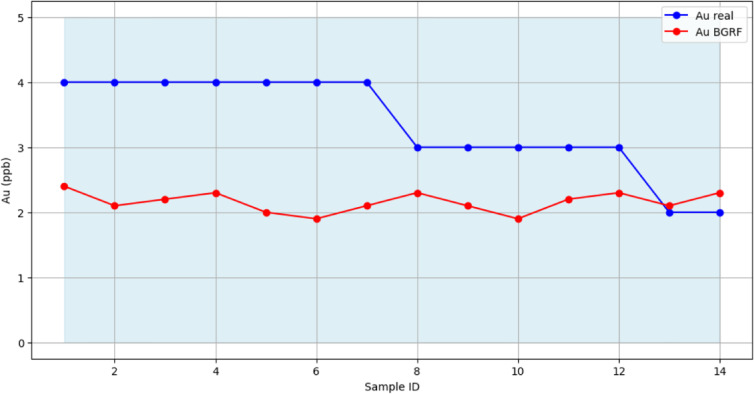
Comparison of real Au and BGRF output with 95% confidence interval for HCD.

The posterior median estimates for censored samples are presented in Fig. 9, showing both the observed Au values and the model predictions along with 95% confidence intervals. The results demonstrate that BGRF effectively preserves the true spatial distribution and local variations of the data, while providing probabilistic predictions for censored samples.

The scientific advantage of BGRF lies in its ability to model not only the expected mean values but also the spatially structured variability and inter-element dependencies. Incorporating both the spatial coordinates and the Cu element, which is highly correlated with Au, allows the model to generate more realistic representations of the background geochemical population. Standardization further ensures stable MCMC sampling and reliable posterior predictions, enhancing the credibility of the results. BGRF provides accurate probabilistic predictions for censored gold values while faithfully preserving the spatial and geochemical patterns of the background population, offering a robust foundation for subsequent geochemical analyses.

### RF modeling of spatial BGRF with covariate

In this study, a RF model was implemented to predict gold concentration using HCD that had been corrected via the spatial BGRF covariate approach. Initially, censored gold values (14 samples) were replaced with the BGRF posterior median estimates, resulting in a complete dataset of 103 samples. In this study, uncertainty was explicitly quantified only in the BGRF stage, where posterior sampling provides full predictive distributions and 95% credible intervals for censored observations. In contrast, the subsequent RF model operates as a deterministic, non-Bayesian learner and therefore does not propagate these posterior uncertainties. To ensure compatibility between the two stages, the RF was trained solely on the posterior median estimates derived from the BGRF output, meaning that only point estimates not their associated uncertainty were carried forward into the machine-learning step.

**Fig. 10:**
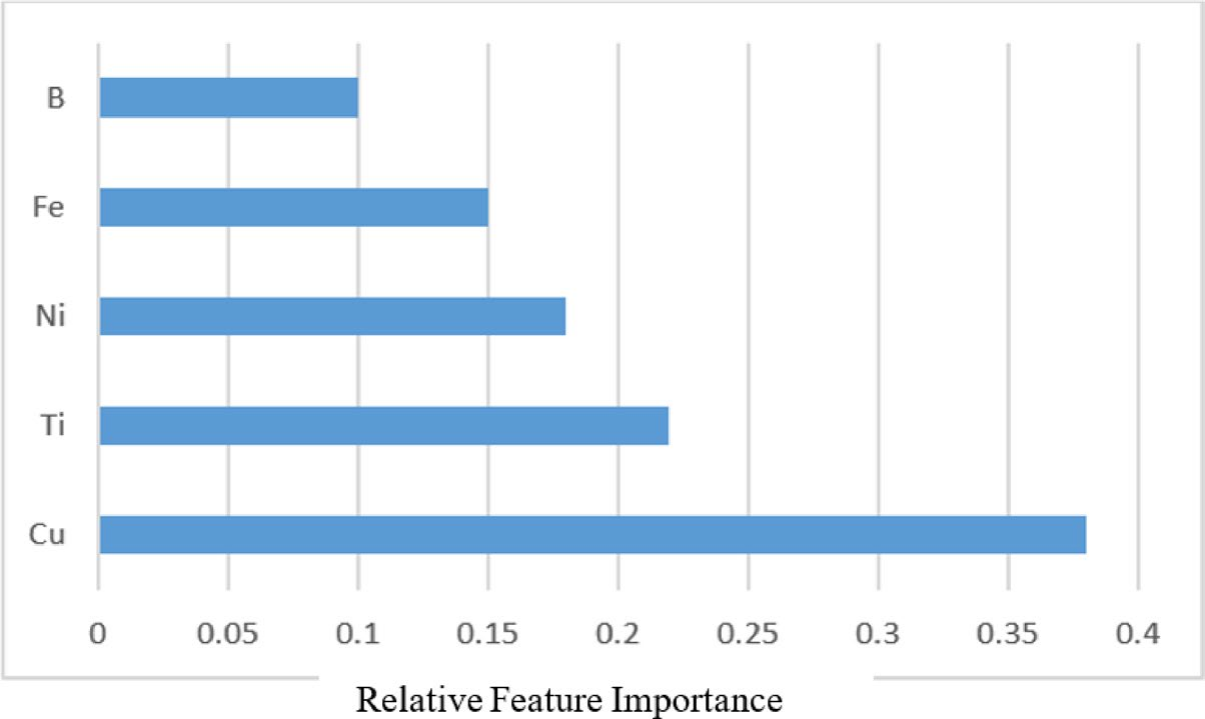
Relative feature importance of the five predictor elements in the RF model.

This step allowed the model to be trained on complete data and mitigated the effects of missing values. Feature selection was performed based on the correlation with the target variable, and five elements Cu, Ti, B, Ni, and Fe exhibiting the highest correlation with Au were selected as model inputs. Copper had the most significant contribution to improving model performance. Prior to model training, all features and Au values were numerically encoded and standardized. To prevent over prediction in low concentration ranges, a logarithmic transformation was applied to the Au values. This transformation reduced positive bias and enhanced the model’s ability to capture the true underlying patterns in the data. Figure 10 illustrates the relative importance of the five predictor elements (Cu, Ti, Ni, Fe, B) in the Random Forest model for Au prediction. Copper exhibits the highest contribution, followed by Ti and Ni, whereas Fe and B provide comparatively lower influence on model performance.

Preprocessing steps included Z-score standardization and mean imputation for missing values. Subsequently, the RF model was trained using five-fold cross-validation to obtain out-of-fold (OOF) predictions for robust performance assessment. In this study, a five-fold cross-validation scheme was adopted. Given the limited size of both the background training dataset (103 samples) and the hypothesized censored subset (14 samples), using k = 5 provides a more stable bias–variance trade-off compared to higher fold numbers. Increasing k to 10 would substantially reduce the training set in each iteration and is known to increase the variance and instability of tree-based models, particularly in small geochemical datasets. Therefore, k = 5 offers a robust and methodologically appropriate choice for generating reliable out-of-fold predictions in the low-concentration range.

To prevent information leakage and to obtain an unbiased estimate of model performance, out-of-fold (OOF) predictions were employed in this study. In the five-fold cross-validation procedure, each sample was predicted only by the model that had not been trained on that specific observation. Accordingly, in each iteration, one fold was held out as the validation set, while the remaining four folds were used for training; predictions for all samples in the validation fold were then generated as OOF outputs. This ensures that every sample is predicted exactly once in a “never-seen” condition, thereby providing an unbiased estimate of predictive behavior. Given the importance of censored samples (HCD) in the evaluation process, their distribution across the folds was conducted in a stratified manner to maintain stable proportions and to ensure that each censored sample was strictly excluded from the training set during the iteration in which it was evaluated. The complete set of OOF predictions was subsequently used for calibration and for comparing the performance of the proposed models.

**Fig. 11:**
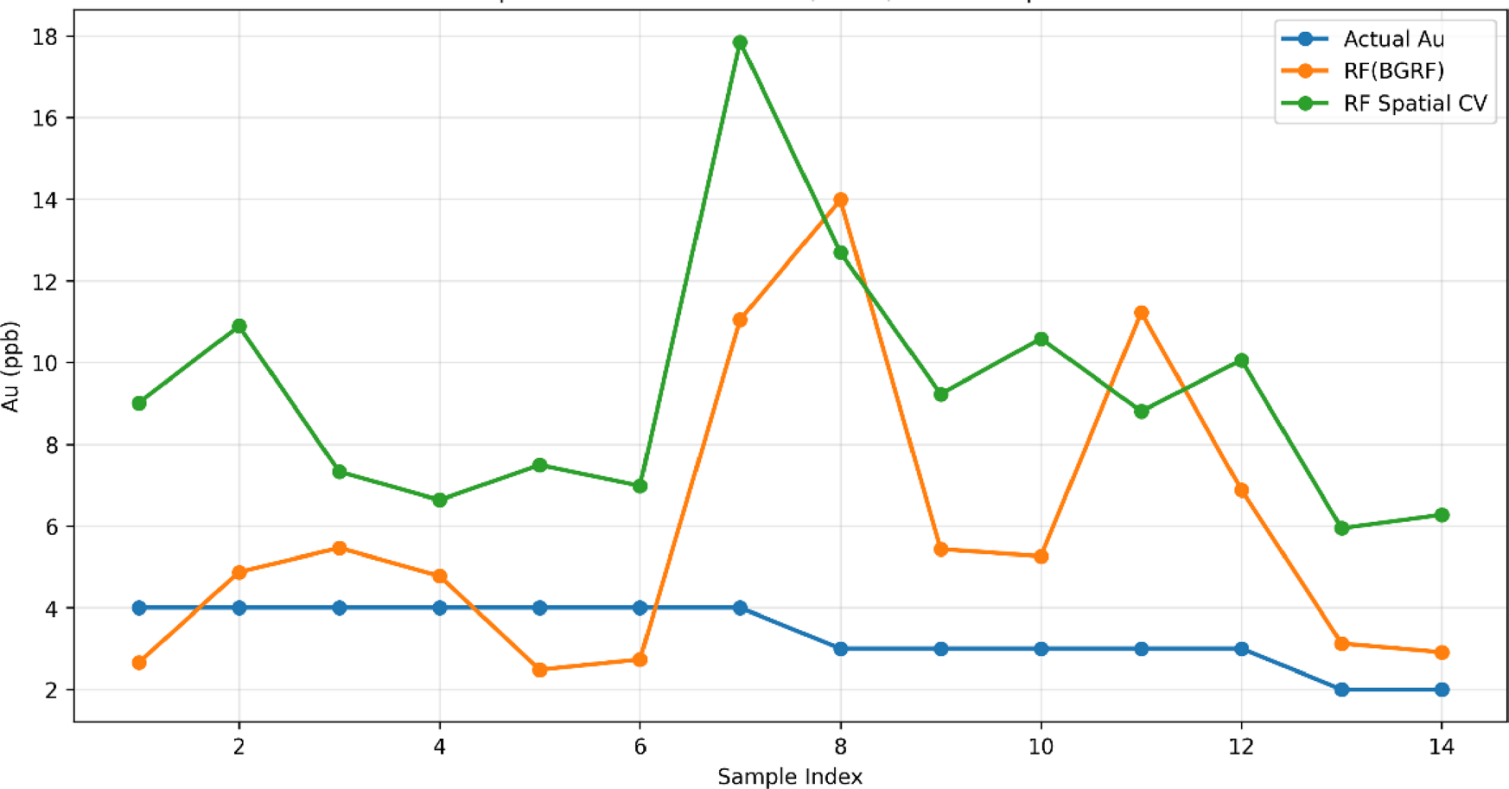
Comparative accuracy of RF and RF–BGRF models in reconstructing censored Au.

To evaluate the extent to which the hybrid RF–BGRF model improved the estimation of censored values and produced predictions closer to the true Au concentrations, an independent comparison with the Random Forest model alone was conducted. In this analysis, the RF Spatial Cross-Validation (CV) procedure was implemented. Unlike conventional random k-fold cross-validation, this method partitions the dataset into spatially coherent folds based on geographical proximity rather than random sampling. Samples that are spatially close to each other are grouped within the same fold to prevent spatial autocorrelation leakage, whereby validation points would otherwise benefit from information provided by nearby training points. Given the inherent spatial dependency in geochemical datasets, random cross-validation yields overly optimistic performance estimates because test samples indirectly inherit information from spatially adjacent training samples. Spatial CV circumvents this issue by ensuring genuine geographical separation between training and validation subsets, providing a more realistic assessment of the model’s predictive capability for truly unseen spatial regions. Accordingly, RF Spatial CV serves as an appropriate benchmark for evaluating the added value of the BGRF component in refining RF predictions. The comparison results illustrate (Fig. 11) that the RF model without BGRF-generated corrected values exhibits substantial fluctuations and frequently overestimates Au concentrations across several samples, particularly samples 7, 8, 9, and 12. This pattern reflects the inability of RF alone to reconstruct the spatial structure and low-grade variability of censored observations. Near the limit of detection, the model suffers from systematic positive bias and fails to accurately represent the subtle background variations.

In contrast, the combined RF-BGRF curve demonstrates that integrating BGRF-derived estimates into the RF model substantially reduces this bias, leading to predictions that consistently align more closely with the true observed values. This improvement is especially pronounced among low-grade samples (1–6 and 10–14), where RF-BGRF avoids the extreme oscillations seen in RF Spatial CV and successfully reproduces the natural geochemical variability of the background population. The enhanced stability of RF-BGRF confirms the corrective role of the BGRF model. By leveraging spatial structure and the strong Au–Cu relationship, BGRF provides reliable initial estimates that effectively constrain the search space of RF, preventing unrealistic predictions. Interpretation of the comparison figure confirms that the RF–BGRF hybrid model not only produces smoother and more accurate predictions but also reconstructs low-grade spatial patterns more faithfully than RF alone. This finding demonstrates that incorporating a Bayesian spatial model as a preparatory estimation step is essential for reducing bias, improving stability, and enhancing generalizability when modeling censored geochemical data.

Initial predictions demonstrated that the model could capture the general trend of the data; however, a noticeable positive bias remained in the lower concentration range (below 5 ppb). To address this issue, a targeted calibration procedure was applied specifically to the lower range of Au concentrations (5–8 ppb). The scientific rationale for focusing on this range lies in the fact that the highest errors and uncertainties occur near the limit of detection (LOD). Calibrating across the entire dataset would result in high-value samples, which contribute more to the overall variance, dominating the calibration process and leaving the low-range predictions inadequately corrected. Therefore, calibrating based on low concentrations allows targeted adjustment where it is most critical, effectively reducing positive bias in censored samples.

The decision to calibrate specifically within the 5–8 ppb interval stems from the well-documented statistical behavior of non-parametric ensemble models particularly Random Forest (RF) and Gradient Boosting Regression under skewed, left-censored distributions. In such models, sparse observations at the lower end of the concentration range result in terminal nodes with few samples, whose means regress toward the overall dataset mean, a phenomenon described as regression to the mean^31^. This shift systematically inflates predictions near the limit of detection (LOD). For censored datasets, the problem is exacerbated because true sub-LOD values are absent; the model infers its low-range behavior solely from the 5–8 ppb samples, which act as imperfect analogues. Consequently, over-prediction bias is most severe close to the LOD.

Calibrating over the entire dataset would allow high-value samples, with their greater variance, to dominate the adjustment, leaving low-range errors largely uncorrected. Targeted calibration in the 5–8 ppb zone directly addresses the range most similar to censored samples and exhibiting the largest residuals, aligning with classical geochemical recommendations to model background and anomalous populations separately^32^. Empirical support for such local calibration near detection thresholds is reported across disciplines, including groundwater studies^33^ and censored multi-depth geochemical modeling^34^.

**Fig. 12:**
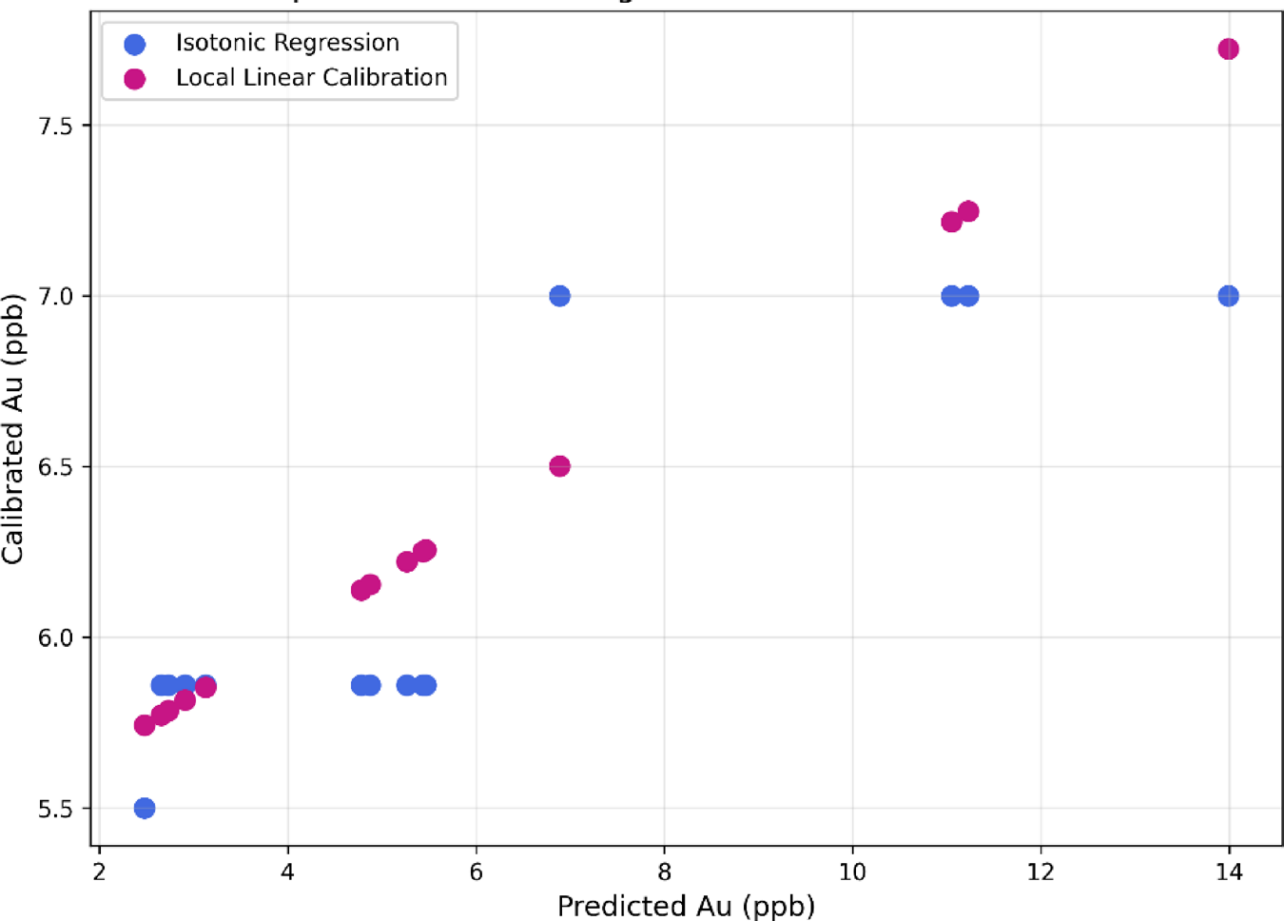
Comparison between linear regression and isotonic regression calibration for low-range Au predictions.

To correct bias and model inconsistency within the lower concentration range, the Rf-BGRF-predicted values were calibrated against the actual measurements within this interval. Two statistical approaches of local linear regression and isotonic regression were implemented for calibration. In the local linear calibration, a simple regression model was fitted between the predicted and observed gold concentrations near the detection limit to estimate the slope and intercept specific to that range. This parametric approach maintains a linear relationship between variables and systematically adjusts the predictions, providing accurate corrections particularly in regions with nearly linear behavior or low noise levels. Conversely, the isotonic method is a non-parametric regression technique based on the assumption of monotonicity between predicted and actual values. Without requiring explicit parameter estimation, it constructs a step-wise monotonically increasing function that follows the ordered data. Originally introduced by^35^ for estimating monotone distributions, isotonic regression has since been widely used in machine learning for bias correction, especially for censored or near-limit data. Recent studies such as Zadrozny and Elkan^36^ and Niculescu-Mizil and Caruana^31^ have shown that the isotonic approach provides flexible nonlinear calibration and maintains monotonic consistency among samples, thereby improving the calibration of nonlinear models such as Random Forest. Figure 12 compares two calibration approaches of linear regression and isotonic regression. The linear calibration generally show slightly higher adjusted values and closer alignment with the predicted axis, whereas the isotonic display a more step-wise monotonic pattern. Overall, the local linear calibration yields marginally superior accuracy and consistency compared with the isotonic regression.

Calibration was performed using a linear relationship between the RF-predicted and observed values within the low-concentration range. Subsequently, a linear scaling procedure was applied to map the maximum calibrated prediction to the LOD (5 ppb). The scaling formula is defined as:

6 $$\:{Au}_{cal-scaled}\left(\mathrm{i}\right)={Au}_{cal}\left(\mathrm{i}\right)\times\:\mathrm{L}\mathrm{O}\mathrm{D}/{Au}_{cal}\left(\mathrm{m}\mathrm{a}\mathrm{x}\right)$$

Where $\:{Au}_{cal}\left(\mathrm{i}\right)$is the calibrated prediction for sample i, $\:{Au}_{cal}\left(\mathrm{m}\mathrm{a}\mathrm{x}\right)$is the maximum calibrated value, and LOD represents the limit of detection.

This transformation enforces a physical upper bound while maintaining the relative spacing among predictions. Statistically, this is equivalent to constraining outputs within a Truncated Gaussian Random Field^7^, where the predictive distribution is clipped to match the observable domain. Such bounded normalization prevents non-physical exceedances and reconstructs the geometric structure of the low-range distribution, yielding residuals symmetric and centered near zero in the scaled model. Similar bounding principles have been implemented via randomized truncations in Gaussian processes to eliminate numerical bias^37^ and via soft bounded mappings in multi-calibration frameworks for censored survival data^38^. Compared with isotonic regression or Platt scaling, the chosen approach is physically interpretable and directly compatible with RF outputs, avoiding distortion of distribution geometry while stabilizing training.

This logic parallels the methodology of LeFrancois & Poeter^39^, who incorporated sub-LOD values as censored observations within a likelihood function, thereby constraining low-range predictions probabilistically. While the present approach does not modify the likelihood directly, its bounded linear mapping applies the same constraint operationally. Likewise, Verbovšek^4^ demonstrated that constant substitution methods such as half of the detection limit (LD-half) and the detection limit divided by the square root of two (LD-rad2) induce positive bias and alter distribution shape, whereas calibration near the LOD preserves the true distribution form a principle leveraged here through model-based correction rather than fixed substitution. This mathematical transformation offers several advantages. It ensures that no predicted value exceeds the LOD, corrects the positive bias in the low-concentration range, and preserves the relative relationships among censored samples. Additionally, by optimizing the distribution of predicted values, this approach enhances model accuracy in low-concentration regions and enables more precise reproduction of the natural variability of gold in geochemical datasets.

**Fig. 13:**
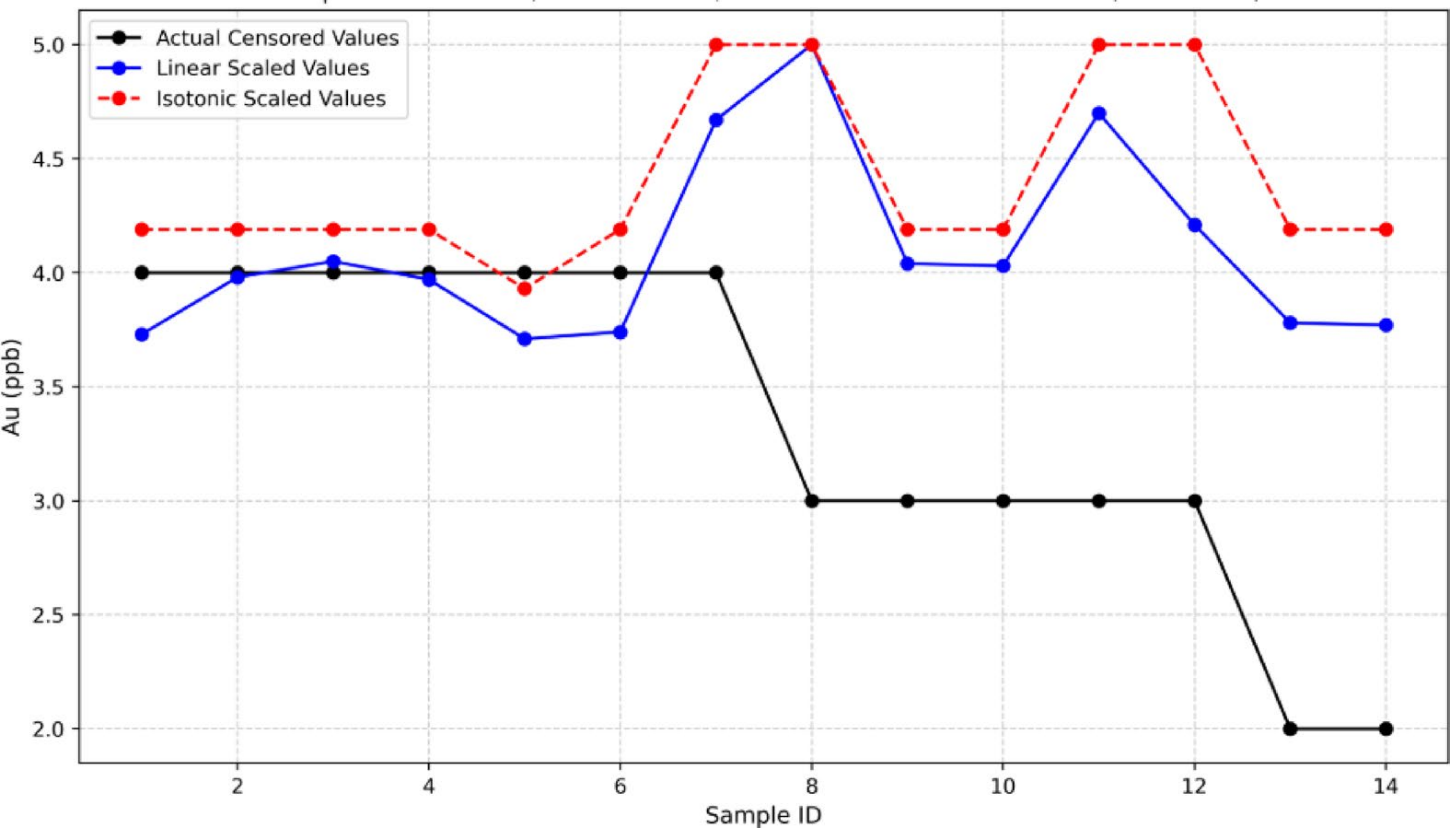
Comparison of calibrated and scaled predictions for sub-LOD gold concentrations using isotonic and linear approaches.

Figure 13 compares the actual censored values with the scaled results derived from isotonic regression calibration and linear regression calibration methods. Both calibration functions were adjusted according to Eq. (6) to bring their maximum predicted values below the detection limit and ensure consistent scaling across the censored range. This bounded scaling procedure guarantees that all calibrated outputs remain less than or equal to LOD while preserving the relative variation among samples. Both processes effectively reduced bias in the near-LOD region; however, the linear regression calibrated scaling approach demonstrated better agreement with the actual concentrations and showed smaller oscillations across samples.

Statistical comparisons indicate that the linear regression calibrated scaling approach achieves the highest accuracy in reproducing the real censored dataset, with mean absolute error around 0.87 and root mean square error close to 1.12, compared with mean absolute error of approximately 1.06 and root mean square error of about 1.35 for the isotonic regression calibrated scaling approach.This indicates lower prediction error and greater numerical stability for the local linear method, while the isotonic regression retains its monotonic continuity but displays a slight overestimation in the lower concentration range. Overall, the results highligh that the linear regression calibrated scaling approach yields more precise and realistic adjustments of sub-LOD geochemical gold data.

According to the results, the linear calibration approach demonstrated better performance than the isotonic calibration method in reproducing the real censored geochemical dataset. Subsequently, the outcomes of the linear regression calibration and linear regression calibrated–scaled models were compared with traditional substitution approaches. The comparison confirmed that the linearly calibrated and scaled models achieved notably higher accuracy, lower prediction error, and more reliable reconstruction of censored gold values than the conventional methods.

**Fig. 14:**
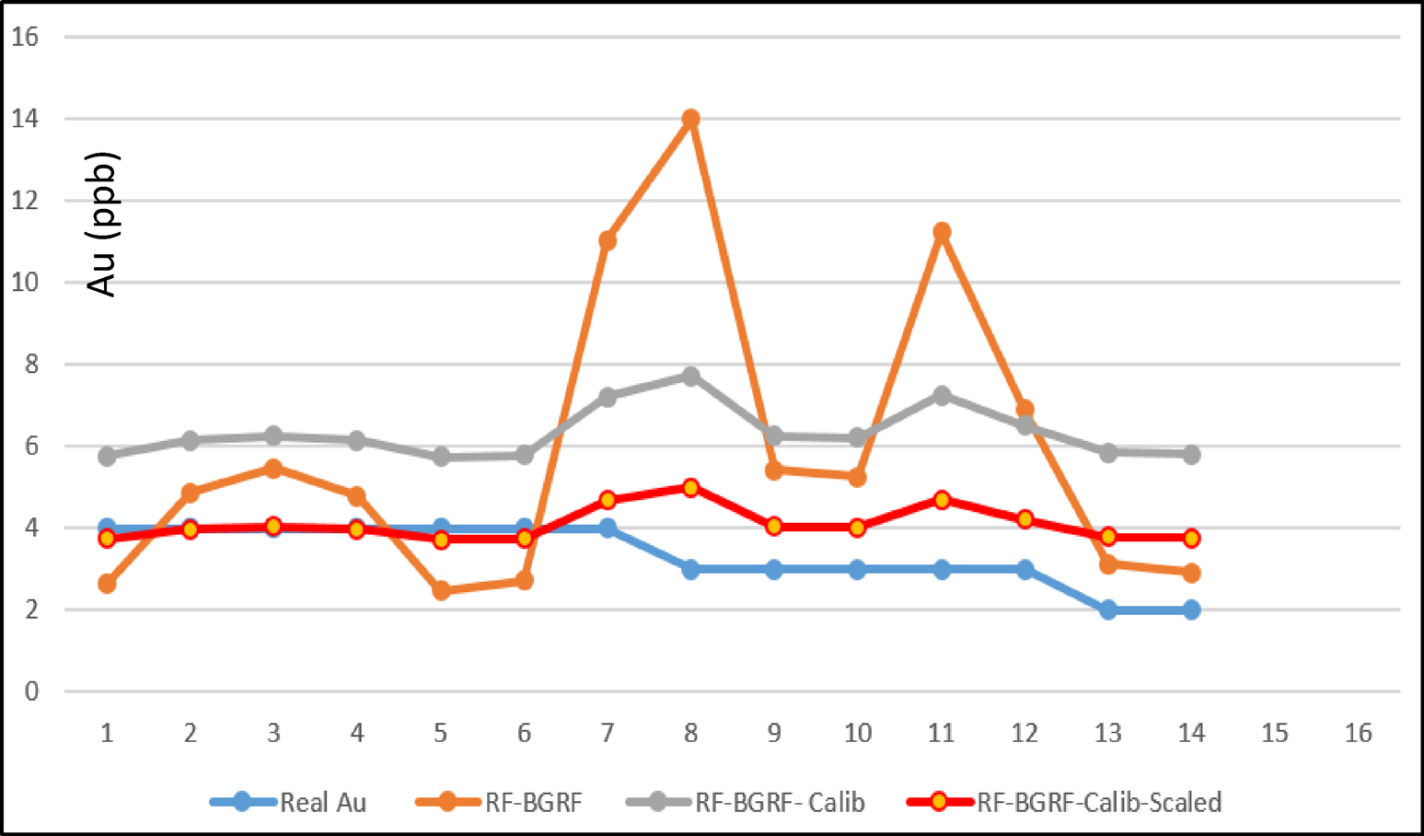
Comparison of real Au concentrations with RF-BGRF predicted, linear calibrated, and scaled values, illustrating the progressive improvement in prediction accuracy across modeling steps.

The results of the three-step modeling process demonstrated progressive improvements in prediction accuracy and error reduction within the lower data range. Calibrated predictions were closer to the observed values than the initial RF outputs, and the scaled predictions showed the highest agreement with the measured data. Boxplots and density plots confirmed that the dispersion of residuals and the mean prediction error were minimized in the final scaled model, providing an accurate reproduction of the true distribution of the data. The results of the three-step modeling procedure are compared together. Figure 14 shows the observed gold values alongside the predictions from the RF model, the calibrated values, and the scaled values. As can be seen, the calibrated predictions are closer to the observed data compared to the initial RF outputs, while the scaled predictions exhibit the best alignment with the actual measurements.

**Fig. 15:**
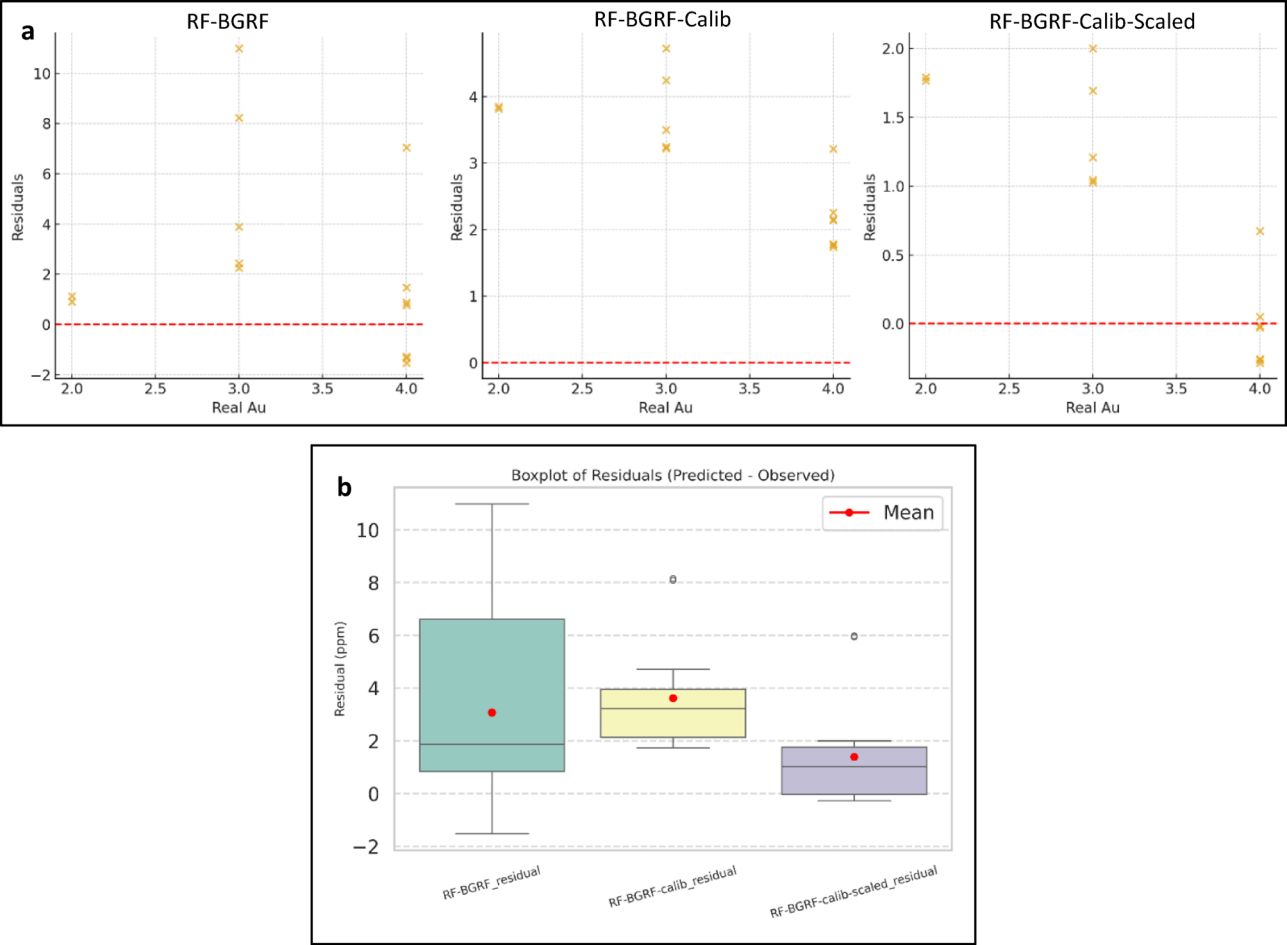
Boxplots of residuals for RF-BGRF, linear calibrated, and scaled predictions, showing the reduction in error dispersion and mean residual in the final scaled model.

**Fig. 16:**
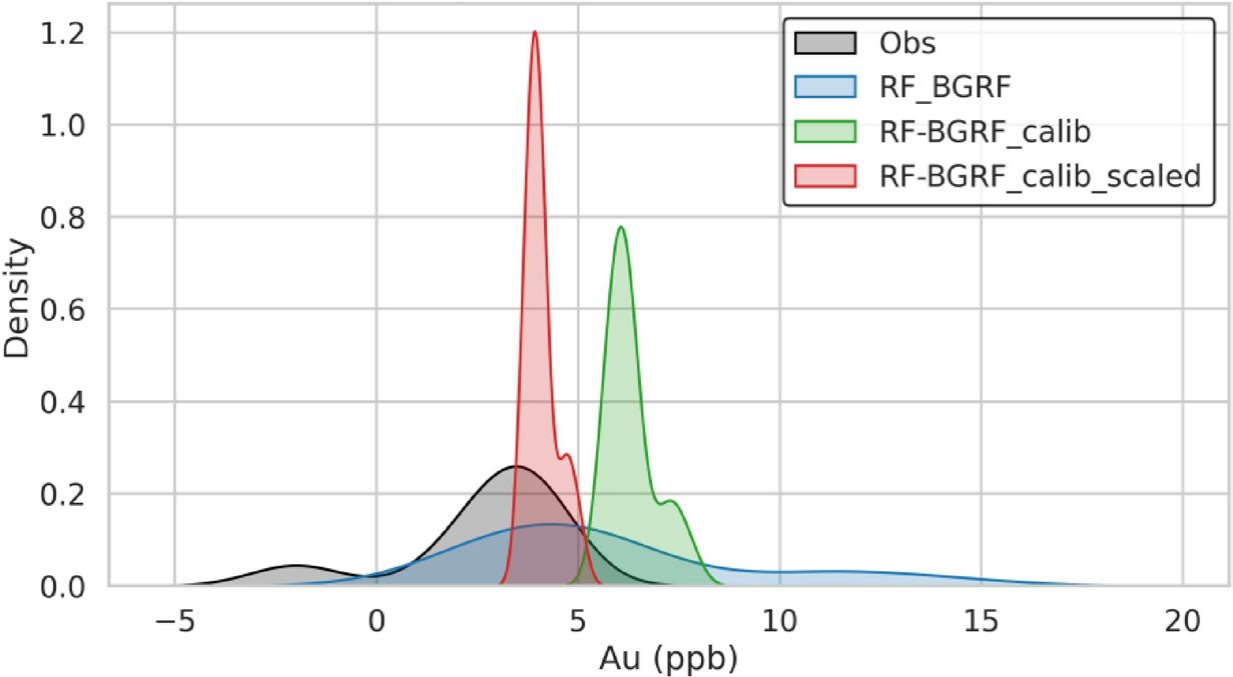
Density plots of observed Au versus predicted values for RF-BGRF, linear calibrated, and scaled models, demonstrating improved reproduction of the true distribution and correction of positive bias in low-concentration values.

Figure 15a illustrates the distribution of residuals for the three modeling approaches of RF-BGRF, RF-BGRF-Cal, and RF-BGRF-Cal -Scaled. A clear systematic reduction in prediction error is evident across the modeling steps. The initial RF-BGRF model exhibits a wide residual spread and a pronounced positive bias, indicating a consistent overestimation of gold concentrations across most sampling points. The application of statistical calibration in the RF-BGRF-Cal model reduces the residual range and concentrates the residuals closer to zero, although localized deviations remain. In contrast, the final RF-BGRF-Cal-Scaled model demonstrates residuals that are tightly constrained, symmetrical, and centered on zero, reflecting substantially improved predictive accuracy and stability. This progressive improvement in the statistical behavior of the models highlights the effectiveness of the calibration and linear scaling procedures in correcting bias, reducing prediction errors, and enhancing concordance with the observed geochemical data. Figure 15b presents boxplots of the residuals for the three scenarios, indicating that both the variability and the mean error are minimized in the final scaled model, demonstrating a clear improvement in predictive consistency. Figure 16 shows the density plots of the observed and predicted values for all three cases, confirming that the linear scaling procedure more accurately reproduces the true distribution of the data and corrects the overestimation bias in the lower concentration range.

**Fig. 17:**
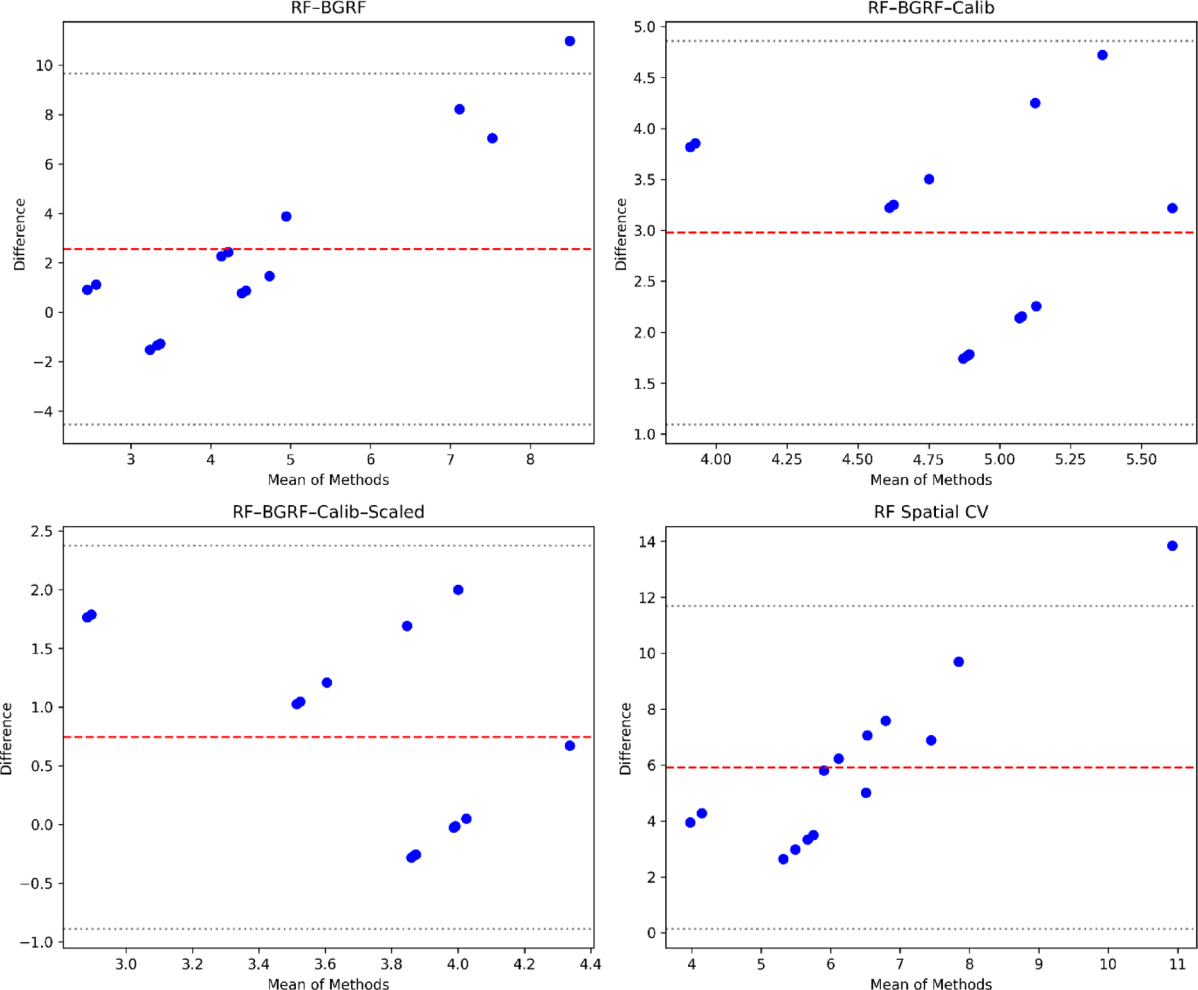
Bland–Altman comparison of four modeling strategies for reconstructing censored Au values.

Table 2 presents the error metrics obtained from applied approaches. The error statistics reveal substantial differences in predictive performance among the four evaluated methods for the hypothesized censored Au samples. The initial RF–BGRF model shows moderate accuracy (MAE = 3.15; RMSE = 4.44) but exhibits a persistent positive bias (2.56), indicating systematic overestimation near the detection limit. Applying calibration to the low-concentration range (RF–BGRF–Calib) reduces overall error, particularly RMSE, though the mean bias remains high due to the narrow calibration interval. The final RF–BGRF–Calib–Scaled model achieves the best performance with a marked reduction in both MAE (0.86) and RMSE (1.12), and a substantially lower bias (0.74), confirming the effectiveness of targeted calibration and scaling in correcting LOD-related distortions. In contrast, the RF Spatial CV baseline yields the largest errors (MAE = 5.92; RMSE = 6.61) and the strongest positive bias, demonstrating limited ability to reconstruct low-grade Au variations. Overall, the calibrated and scaled RF–BGRF approach provides significantly more accurate and unbiased predictions for censored geochemical data. This marked improvement demonstrates that scaling not only mitigates the effect of large differences in data range but also enhances numerical stability and model training, resulting in more accurate predictions. The Bland–Altman plots (Fig. 17) illustrate the agreement between observed Au concentrations and predictions from four modeling strategies. The RF–BGRF and RF–BGRF–Calib models exhibit moderate positive bias, while the final RF–BGRF–Calib–Scaled approach shows the smallest mean difference and the narrowest limits of agreement. In contrast, the RF Spatial CV method demonstrates large systematic deviations, confirming its poor suitability for estimating Au values near the detection limit.

**Table 2:** Performance metrics of the four prediction scenarios for censored Au samples, including MAE, RMSE, systematic bias, sample size (*n* = 14), and 95% bootstrap confidence intervals for all error metrics.

Model	MAE	RMSE	Bias
RF-BGRF	3.15	4.44	2.56
RF-BGRF-Calib	2.98	3.13	2.98
RF-BGRF-Calib-Scaled	0.86	1.12	0.74
RF spatial CV	5.92	6.61	5.92

**Fig. 18:**
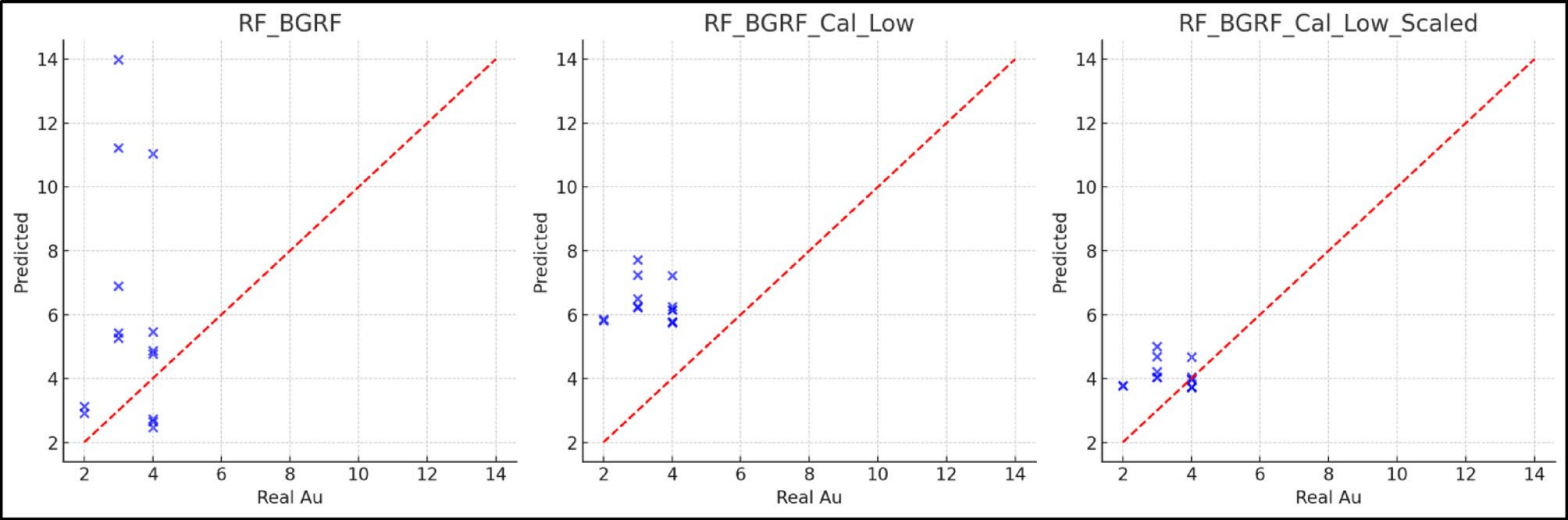
Scatter plots of predicted values versus actual Au concentrations for three proposed models; the RF-BGRF-Cal-Scaled scenario indicates improved predictive accuracy and reduced bias.

Figure 18 illustrates the progressive improvement in predictive accuracy of the RF-BGRF models by comparing the predicted gold concentrations with the observed values. In the initial RF-BGRF model, data points are systematically positioned above the ideal line (y = x), indicating significant overestimation and the presence of a positive bias in the model outputs. With the application of statistical calibration in the RF-BGRF-Cal model, the dispersion of points is reduced and relative agreement with the observed values is achieved, although some localized deviations persist. In the final model following linear scaling (RF-BGRF-Cal-Scaled), points are tightly clustered around the ideal line, reflecting successful bias correction, a reduction in structural prediction errors, and enhanced statistical concordance between predicted and observed values. This trend highlights the effectiveness of the calibration and normalization procedures in improving model quality and reliability, particularly in geochemical applications.

### Machine learning vs. classical substitution approaches

**Fig. 19:**
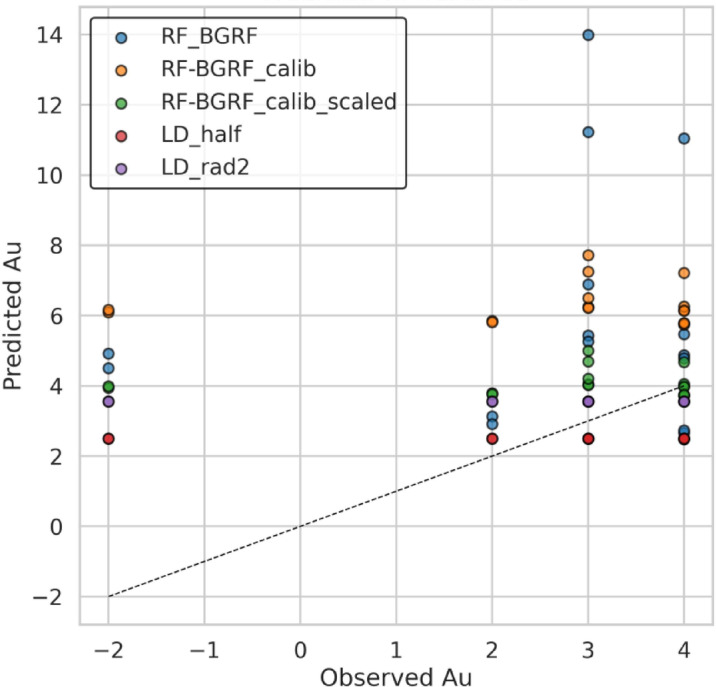
Comparison of predicted values versus observed Au concentrations for five applied approaches, illustrating progressive improvements in predictive accuracy and bias reduction.

In this section, the performance of the RF machine learning method combined with the Bayesian Gaussian algorithm (RF-BGRF) and its refined versions was compared with two traditional approaches for handling censored data. The conventional methods include substituting censored values with LD-half and LD-rad2, which are widely used in geochemical studies due to their simplicity and ease of application. Figure 19 illustrates the relative performance of five different models in predicting the concentration of gold for HCD. In the baseline RF-BGRF model, a broad dispersion and systematic deviation from the ideal line (y = x) are observed, indicating systematic overestimation at higher values and poor reconstruction of the true data distribution. The RF-BGRF-Calib model reduced dispersion and showed closer agreement with the ideal line, resulting in improved prediction accuracy. The final RF-BGRF-Calib-Scaled model exhibited the highest overlap with the ideal line and the least dispersion, highlighting successful bias correction and enhanced statistical precision. In comparison, the traditional LD-half and LD-rad2 models, although showing reasonable agreement at lower values, demonstrated weaker accuracy in higher ranges and lagged behind the refined RF-BGRF-Calib-Scaled model. These findings indicate that calibration and scaling within RF-BGRF not only reduce prediction errors and correct bias but also significantly improve its performance over threshold-based substitution methods.

One of the key findings relates to six samples with relatively high censored values. In this case, the RF-BGRF-Calib-Scaled model achieved an exceptionally low mean relative error of about 0.003, whereas this metric for the LD-half and LD-rad2 approaches was approximately 0.37 and 0.11, respectively. This substantial difference highlights the superior capability of the machine learning approach in reconstructing higher censored values and preventing systematic bias. Such an ability is crucial for preserving and identifying geochemical patterns and relationships, and unlike simple substitution methods, it does not distort inter-element relationships or geochemical parameters, particularly when censored data are abundant and play a significant role in multivariate analyses.

In this context, correlation analysis further confirmed that machine learning models preserve inter-variable relationships more effectively than traditional substitution methods. For example, the correlation coefficient between actual Au and Cu concentrations in HCD was 0.36, and the predicted data from the RF-BGRF-Calib-Scaled model yielded a correlation coefficient of 0.23. This slight difference demonstrates that the applied predictive model successfully captured the underlying inter-element relationships and geochemical patterns. In contrast, traditional substitution methods result in zero variance for substituted values, rendering correlation analysis essentially meaningless.

**Fig. 20:**
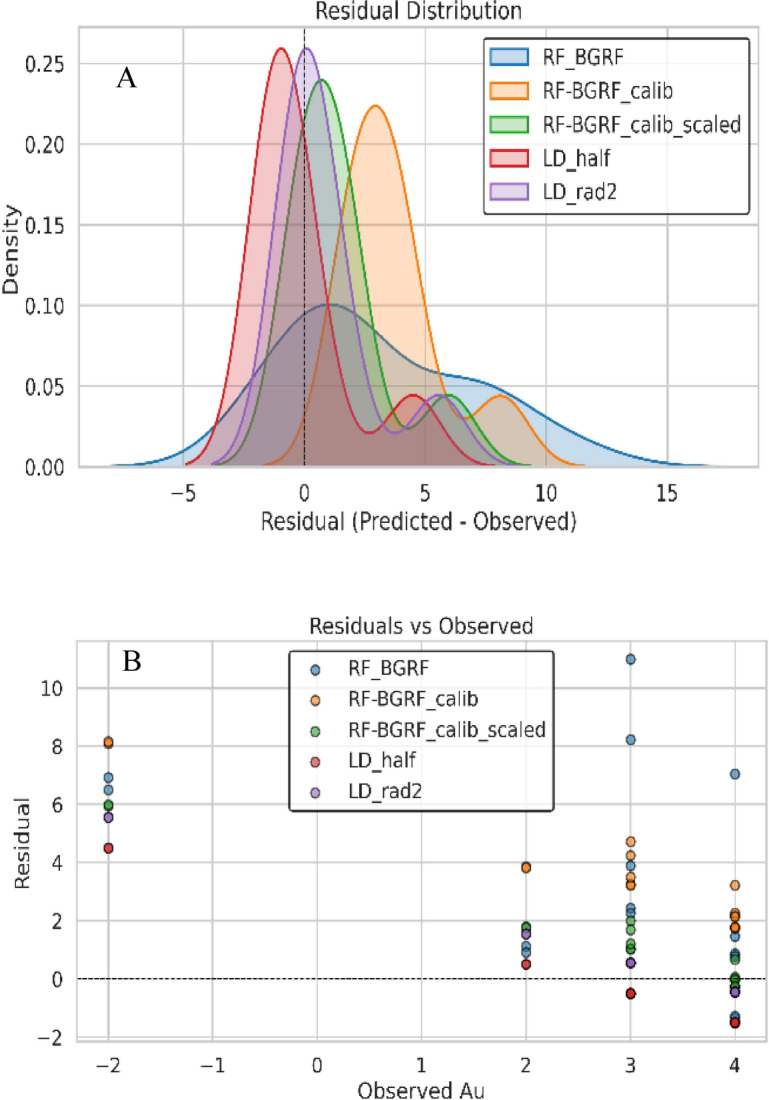
Comparative residual analysis of gold prediction models: (**A**) density distributions of residuals; (**B**) residuals plotted against observed Au values, highlighting performance differences among the evaluated methods.

Figure 20 compares the relative performance of five gold prediction models based on residual analysis. In the residual density plot (A), the initial RF-BGRF model exhibits a skewed distribution with a long tail toward positive values, indicating systematic positive bias and a tendency to overestimate concentrations. After calibration in RF-BGRF-calib method, the residuals become more centered, and in the final RF-BGRF-calib-scaled model, they converge into a narrow and symmetric distribution around zero, reflecting both reduced dispersion and bias correction. In contrast, the traditional LD-half and LD-rad2 models show distributions with negative peaks and left-skewness, suggesting structural underestimation in their predictions. Among the substitution-based approaches, however, all error indices and residual patterns confirm that LD-rad2 method yields more reliable results than LD-half method. In the residuals versus observed values plot (Fig. 20B), the RF-BGRF model generates predominantly positive and scattered residuals, while in RF-BGRF-calib method, the residuals move closer to the zero line. The final RF-BGRF-calib-scaled model produces residuals concentrated tightly around zero, with no discernible dependency on observed values. By contrast, both LD-half and LD-rad2 methods exhibit residuals not only biased toward negative values but also clearly dependent on observed concentrations, indicating heteroscedasticity and weak generalization ability.

Overall, this comparison clearly demonstrates that the calibration and scaling process in RF-BGRF-calib-scaled method not only reduces predictive error but also improves the error structure, thereby enhancing the model’s statistical robustness, stability, and reliability.

**Fig. 21:**
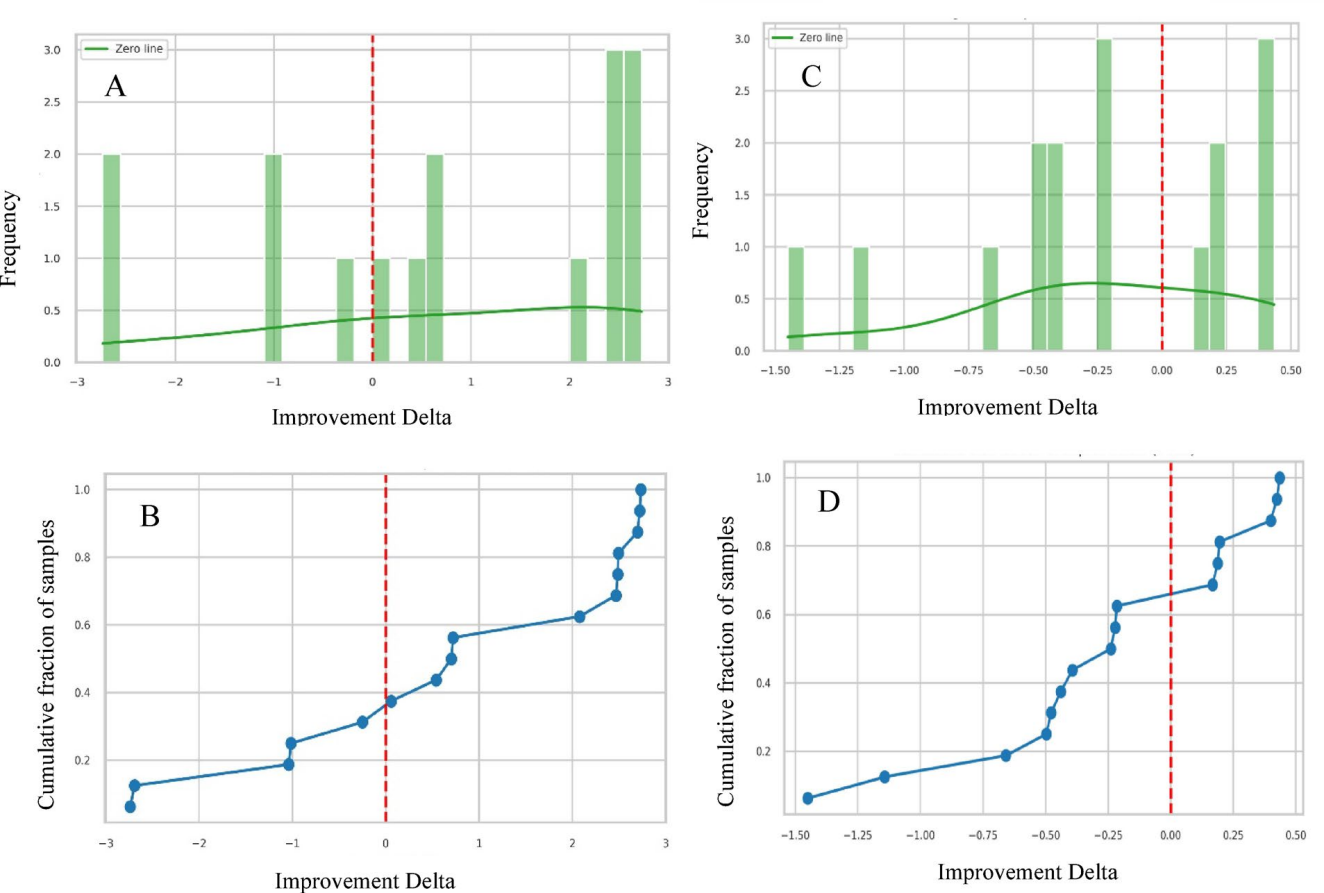
Histograms and cumulative distribution plots of the improvement Delta for RF-BGRF-calib-scaled method compared to traditional methods of LD-half (**A**, **B**) and LD-rad2 (**C**, **D**).

**Fig. 22:**
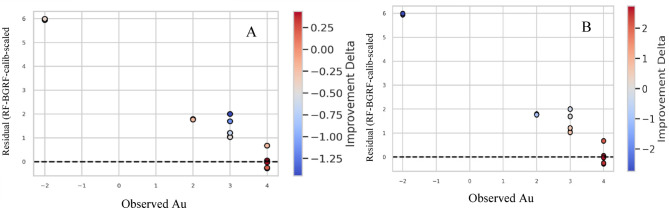
Improvement delta analysis of RF-BGRF-calib-scaled method vs. LD-rad2 (**A**) and LD-half (**B**).

Figures 21 and 22 illustrate the performance of the machine learning RF-BGRF-calib-scaled model in comparison with the two common substitution methods, LD-half and LD-rad2, based on standardized residual analysis and the Delta index. The Delta index is defined according to the following equation:

7 $$\:{\Delta\:}=\mid\:{Au}_{(LD-half)or(\mathrm{L}\mathrm{D}-\mathrm{r}\mathrm{a}\mathrm{d}2)}\text{}-{Au}_{real}\mid\:-\text{}\mid\:{Au}_{(\mathrm{R}\mathrm{F}-\mathrm{B}\mathrm{G}\mathrm{R}\mathrm{F}-\mathrm{c}\mathrm{a}\mathrm{l}\mathrm{i}\mathrm{b}-\mathrm{s}\mathrm{c}\mathrm{a}\mathrm{l}\mathrm{e}\mathrm{d})}\text{}-{Au}_{real}\mid\:\text{}\text{}$$

Positive values of Delta indicate the superiority of the machine learning model over simple substitution methods in reconstructing actual censored values.

Figure 21 provides a statistical comparison of the performance of the RF-BGRF-calib-scaled model in estimating censored gold concentrations. Panels A and B compare the RF-BGRF-calib-scaled model with the LD-half method and show that in 68.75% of the samples, the RF-BGRF-calib-scaled model achieved higher accuracy than the simple substitution approach. The histogram pattern in panel A, which is predominantly concentrated on the positive side of the Delta axis, together with the cumulative curve in panel B that clearly crosses above zero, demonstrates that the RF-BGRF-calib-scaled model was able to capture local variations more accurately and provided a meaningful average improvement (Δ ≈ 0.748). In contrast, panels C and D compare the model with the LD-rad2 method, where only about 37% of the samples exhibited improved performance. Nevertheless, the cumulative curve in panel D indicates that the LD-rad2 approach offers greater stability in estimating mean values, although it performs less accurately in reconstructing spatial details and geochemical anomalies.

Figure 22 evaluates the relative performance of the RF-BGRF-calib-scaled model compared with the two common substitution methods based on standardized residual analysis and the improvement Delta index.

In panel B (comparison with LD-half), the color-coded points reveal that a substantial portion of the samples exhibit positive delta values, particularly in the range above 2 ppm. This indicates that the RF-BGRF-calib-scaled model reduced prediction errors relative to LD-half method and represented spatial variability with greater precision. Residuals in this panel are distributed mostly near zero, with no apparent dependence on observed values, reflecting statistical stability and the absence of systematic bias. In contrast, panel A (comparison with LD-rad2) shows that although some samples demonstrate improvement, a considerable portion exhibit negative delta values. This suggests that the LD-rad2 method may yield more stable mean estimates under certain conditions but lacks accuracy in reconstructing local variations and identifying geochemical anomalies. Furthermore, the higher residual dispersion and the presence of dependence patterns on observed values in this panel indicate potential heteroscedasticity and reduced generalizability of the LD-rad2 method.

The obtained results show that the RF-BGRF-calib-scaled model not only significantly reduces prediction errors but also outperforms traditional substitution methods in reconstructing spatial structures and geochemical relationships. These advantages highlight the efficiency and reliability of this model for applications in mineral exploration and geochemical data analysis.

Although traditional substitution methods provide a rapid and preliminary estimation of censored data, their inherent nature leads to reduced variance, excessive smoothing, and the loss of part of the natural signal. Consequently, while the overall mean of the dataset is approximately preserved, the true statistical and spatial patterns are not accurately reflected. In contrast, the machine learning based approach, using RF-BGRF and its calibrated and scaled variants, yields more dynamic and realistic estimates. Evaluation of error indices revealed that, although differences between this learning-based method and LD-rad2 are not always substantial, distributional analyses such as the Delta index and error histograms demonstrated that the RF-BGRF-Calib-Scaled model represents background variability with higher accuracy.

This capability is particularly important in geochemical exploration, where even minor fluctuations in background ranges may indicate significant geological processes. The findings suggest that the choice of substitution method should be aligned with the research objective. When only a rapid approximation of mean values is required, traditional LD-based methods may suffice. However, when the goal is to preserve natural variability, reconstruct data with higher fidelity, and control bias in censored values, the machine learning approach using the modified RF-BGRF method offers a measurable advantage. This performance difference becomes more evident when the proportion of censored data is high or when censored values are concentrated in the upper part of the distribution. Despite the remarkable efficacy of the proposed framework in predicting censored gold data in this study, it is important to acknowledge its potential limitations. The model’s performance, though robust, may be contingent upon specific geochemical correlations between elements and the geological context of the studied area, which necessitates further investigation for direct generalizability to entirely different elements or geological environments. Furthermore, the selection of a single covariate in the BGRF model and the sensitivity of the fractal method to defined thresholds highlight clear avenues for future research aimed at enhancing the framework’s stability and comprehensiveness. These considerations not only clarify the model’s scope of application but also open up horizons for its future development and adaptation to more complex geochemical scenarios.

The following considerations delineate the primary limitations of the present study and define the scope within which the proposed approach should be interpreted. The reported error metrics are derived from a limited number of hypothesized censored samples (*n* = 14), reflecting the intrinsic data scarcity in the low-concentration range. Within this constraint, the results provide a focused and conservative evaluation of model behavior near the detection limit rather than a comprehensive assessment across the full concentration spectrum. Consequently, the reported performance metrics are intended to characterize predictive behavior within this specific domain and should not be interpreted as fully representative of overall model generalization. Moreover, the effectiveness of the proposed framework is inherently influenced by the statistical and geological structure of the input data, including distributional characteristics, sample size, data quality, and spatial or geological context. As such, variations in predictive accuracy and error magnitude may be expected when applying this methodology to datasets from other regions with differing geochemical associations or geological settings. While the findings demonstrate the validity and practical utility of the proposed approach for the studied case, direct transferability to other data conditions should therefore be undertaken with appropriate caution.

## Conclusion

This study addressed the challenge of predicting censored geochemical data by developing a calibrated and scaled Random Forest–Bayesian Gaussian Random Field framework (RF-BGRF-calib-scaled). To further validate the framework, 14 samples with Au concentrations below 5 ppb were treated as hypothetically censored data (HCD), allowing a more rigorous assessment of model performance against their actual measured values. The findings demonstrate that this approach consistently outperforms both conventional substitution techniques and the uncalibrated baseline RF-BGRF model. When evaluated against the uncalibrated RF-BGRF predictions, the calibrated and scaled version produced narrower residual distributions, reduced heteroscedasticity, and closer agreement with observed data. This underscores the critical role of calibration and scaling in correcting systematic overestimation and enhancing the interpretability of machine learning outcomes in geochemical applications. Relative to the LD-half substitution method, the model achieved positive improvements for 68% of samples (mean Δ ≈ 0.748), reflecting its capability to reduce systematic bias and more accurately reconstruct censored values. Although only 37% of samples improved compared to the LD-rad2 method, residual diagnostics confirmed that the RF-BGRF-calib-scaled model preserved spatial variability and geochemical structure more effectively, whereas LD-rad2 tended to yield stable yet less informative estimates. Notably, for six samples with relatively high censored values, the RF–BGRF–Calib–Scaled model achieved an exceptionally low mean relative error (0.003), in contrast to the substantially higher errors observed for LD-half (0.37) and LD-rad2 (0.11), thereby underscoring its superior capacity to reconstruct higher-end censored measurements. A notable strength of the proposed method lies in its ability to maintain natural geochemical patterns and stable inter-element correlations, which are often compromised by simplistic substitution rules. Furthermore, it preserved the inherent variability of the dataset and clearly distinguished censored from uncensored values, thereby avoiding artificial homogenization. Importantly, the integration of fractal background separation played a critical role in suppressing over prediction, isolating true geochemical signals, and enhancing the accuracy of censored value reconstruction.

The principal innovation of this work is the integration of probabilistic spatial modeling with machine learning, coupled with calibration and scaling procedures tailored to censored datasets. By minimizing prediction error, mitigating systematic bias, and preserving multivariate geochemical relationships, the RF-BGRF-calib-scaled model provides a robust and defensible alternative to substitution-based approaches and uncalibrated machine learning methods. This framework offers not only a reliable tool for geochemical data analysis but also a valuable methodological advancement for mineral exploration and environmental geochemistry.

## Data Availability

The datasets analyzed during the current study are not publicly available due to confidentiality agreements and data ownership restrictions imposed by the private mining company, but are available from the corresponding author on reasonable request.
